# Multi-Mechanism Artificial Lemming Algorithm for Global Optimization and Color Multi-Threshold Image Segmentation

**DOI:** 10.3390/biomimetics11030161

**Published:** 2026-02-28

**Authors:** Liang Tao, Lingzhi Li, Fan Lu

**Affiliations:** 1 School of Future Science and Engineering, Soochow University, Suzhou 215222, China; 20245258020@stu.suda.edu.cn (L.T.); fanluciam@gmail.com (F.L.); 2 Key Laboratory of General Artificial Intelligence and Large Models in Provincial Universities, Soochow University, Suzhou 215222, China

**Keywords:** artificial lemming algorithm, swarm intelligence, multi-threshold image segmentation, CEC2017, global optimization

## Abstract

Color multi-threshold image segmentation is a non-convex, gradient-free global optimization problem. The number of decision variables increases with the number of thresholds, leading to a rapid expansion of the search space and increased computational complexity. To address this problem, this paper proposes a Multi-Mechanism Artificial Lemming Algorithm (MALA). When applied to color multi-threshold image segmentation, the original Artificial Lemming Algorithm (ALA) suffers from an imbalance between exploration and exploitation, excessive reliance on the current best solution, and rigid boundary handling, which may lead to premature convergence and suboptimal threshold selection. MALA integrates three lightweight yet structurally enhancement mechanisms to enhance the stability of the exploration–exploitation process, population-level guidance, and boundary-handling behavior. To verify its general optimization capability, MALA is evaluated on the CEC2017 benchmark suite, where it shows competitive convergence behavior and improved objective values compared with ALA and representative baseline algorithms. Furthermore, segmentation experiments on six benchmark images using Otsu’s criterion show that MALA attains competitive fitness values and generally higher PSNR, SSIM, and FSIM metrics. These results suggest that MALA can serve as a general optimization method with applicability to color multi-threshold image segmentation.

## 1. Introduction

With the continuous development of computer vision, multi-threshold image segmentation has been widely applied in many fields, such as medical image analysis [1,2], remote sensing image interpretation [3], object detection [4], and scene understanding [5]. In practice, images often exhibit complex textures, varying lighting conditions, and inhomogeneous distribution of target regions [6]. As a result, in color multi-threshold image segmentation, the number of decision variables increases with the number of thresholds, which causes the search space to expand rapidly and makes the problem non-convex and gradient-free [7,8,9]. When attempting to exhaustively search all combinations of thresholds or to precisely model this problem, limited computational resources make it difficult to obtain an effective solution within a reasonable time [10,11,12,13]. Consequently, color multi-threshold image segmentation is a challenging global optimization problem that requires efficient and stable meta-heuristic strategies [14].

To address the above optimization challenges in color multi-threshold image segmentation, various bio-inspired optimization algorithms, such as Particle Swarm Optimization (PSO) [15], Salp Swarm Algorithm (SSA) [16], Artificial Bee Colony (ABC) [17], Firefly Algorithm (FA) [18], and Moth–Flame Optimization (MFO) [19], have been widely applied in recent years [20]. However, with the adoption of increasingly complex structures and control strategies, the dependence of these algorithms on parameters has increased significantly, which further aggravates stability and constraint-handling issues in multi-threshold image segmentation [21,22]. In this context, how to improve the stability and robustness of the search process under complex constraints while maintaining a concise structure and controllable parameters remains a critical issue in color multi-threshold image segmentation.

To develop an optimization framework that maintains structural simplicity while providing improved stability under constraints, Xiao et al. [23] proposed the Artificial Lemming Algorithm (ALA). By linking individual movement to energy considerations, ALA simulates lemming behavior during long-distance migration, burrowing, foraging, and evading predators and translates these biologically inspired survival strategies into an effective search mechanism. Compared with many meta-heuristic algorithms that rely on numerous control parameters, ALA features a simple structure, few parameters, and is easy to implement, and has demonstrated competitive performance on a variety of benchmark optimization problems, especially under limited evaluation budgets. However, the ALA still has several practical problems when used for color multi-threshold image segmentation, which is a gradient-free and bound-constrained optimization task. Specifically, the energy-gating mechanism may bias individuals toward a single behavioral branch during the middle and late iterations, leading to unstable transitions between exploration and exploitation. Moreover, population guidance that relies solely on the current best solution can be sensitive to noise and local optima. In addition, the direct boundary projection strategy may disrupt well-adapted solution components, thereby inducing boundary clustering or oscillatory behavior. As a result, these limitations may lead to premature convergence and trapping in local optima.

To alleviate these issues and improve the stability and reliability of threshold optimization, while retaining its biologically inspired foundation, this paper proposes a Multi-Mechanism Artificial Lemming Algorithm (MALA). MALA maintains low computational complexity while introducing multiple lightweight and structured enhancement mechanisms. It reduces sensitivity to stochastic fluctuations and boundary-handling effects, thereby improving the stability and adaptability of the search process. The main contributions are summarized as follows:

- An energy-based behavior smoothing mechanism is proposed to enhance transition stability between exploration and exploitation, thereby reducing premature convergence and improving the reliability of threshold selection in multi-threshold image segmentation.
- A population guidance refinement strategy incorporates significant elite solution information, which provides stable guidance at the population level and improves convergence robustness when optimizing multiple thresholds simultaneously.
- A boundary-handling improvement strategy uses a localized repair strategy to address constraint violations, thereby avoiding boundary clustering phenomena and ensuring the feasibility of threshold configurations in color multi-threshold image segmentation.
- MALA is evaluated using the CEC2017 benchmark test suite to verify its general optimization capability, and on color multi-threshold image segmentation problems to demonstrate its practical effectiveness.

The subsequent structure of this paper is as follows: Section 2 first reviews the original ALA and then details the proposed MALA, Section 3 presents the experimental setup and results analysis on the CEC2017 test suite, Section 4 applies the MALA to multi-threshold image segmentation and reports the experimental results, and Section 5 discusses the research conclusions and potential future research directions.

## 2. Multi-Mechanism Artificial Lemming Algorithm (MALA)

### 2.1. The Original Artificial Lemming Algorithm (ALA)

The original ALA balances the search range and precision by mimicking four collective behaviors of lemmings: long-distance migration, digging holes, foraging, and evading predators. Only the baseline operators and the energy-based switching rule that are relevant to the subsequent modifications are introduced in this subsection.

#### 2.1.1. Initialization

*N* is defined as the population size, *i* is the index of an individual in the population, and dim is the dimension of the optimization problem. The search bounds are vectors $\mathrm{lb},\mathrm{ub}\in R^{dim}$, where rand∼U(0,1). The initial population is created by random initialization:

(1) $$x_{i,j}=lb_{j}+\mathrm{rand}\;\cdot \;\left(ub_{j}-lb_{j}\right),\;i=1,\ldots ,N,\;j=1,\ldots ,dim.$$

#### 2.1.2. Energy Factors

To balance exploration (migration/digging) and exploitation (foraging/evading), ALA introduces an energy factor that decreases with the number of iterations. Here, *T* is the maximum number of iterations, and t∈{1,…,T} denotes the current iteration index:

(2) $$E_{i}^{\left(t\right)}=2\;\ln\;\left(\frac{1}{\mathrm{rand}}\right)\theta \left(t\right),\;\theta \left(t\right)=2\arctan\left(1-\frac{t}{T}\right).$$

When $E_{i}^{\left(t\right)}>1$, the algorithm performs exploration; otherwise, it performs exploitation.

#### 2.1.3. Four Behavior Update Operators

At each iteration, ALA updates individual positions by randomly selecting one of four behavior operators according to the current search phase. In the exploration phase, long-distance migration is selected when rand<0.3; otherwise, digging holes is performed; in the exploitation phase, foraging is selected when rand<0.5; otherwise, evading predators is executed.

Since the mathematical formulations of these four behavior update operators are not modified in MALA, their detailed update equations and parameter definitions can be found in the original ALA paper.

#### 2.1.4. Behavioral Gating

If the updated position violates the search bounds, a hard projection is applied:

(3) $${\left[\Pi_{\left[lb,ub\right]}\left(x_{i}^{\left(t+1\right)}\right)\right]}_{j}=\min\left\{\max\left\{x_{i,j}^{\left(t+1\right)},\;lb_{j}\right\},\;ub_{j}\right\},\;j=1,\ldots ,dim.$$

Here, $\Pi_{\left[lb,ub\right]}\left(\;\cdot \;\right)$ denotes the component-wise projection operator that enforces bound constraints. Then, greedy selection is performed between $x_{i}^{\left(t\right)}$ and the repaired candidate ${\tilde{x}}_{i}^{\left(t+1\right)}=\Pi_{\left[lb,ub\right]}\left(x_{i}^{\left(t+1\right)}\right)$.

### 2.2. Proposed Enhancement Modules in MALA

In bound-constrained optimization, the original ALA may become sensitive to three factors. Specifically, it may be affected by stochastic fluctuations around the energy gate, unstable guidance when relying solely on the current best individual, and feasibility repair that may damage well-adapted components. Therefore, MALA retains ALA’s four behavioral operators and introduces three lightweight modules to improve gate stability, guidance stability, and repair stability, respectively.

#### 2.2.1. Logistic Chaos Perturbation Function (LCEF)

In the original ALA, the energy gate is determined by Equation (2), with a decreasing annealing factor. In the mid- and late stages, when the annealing factor becomes small, a single random sample may overly bias some individuals toward one behavioral branch for many iterations (with $E_{i}^{\left(t\right)}>1$ or $E_{i}^{\left(t\right)}\leq 1$ persisting in the long term). This exacerbates the imbalance between exploration and exploitation, reinforcing the influence of a single random sample on the search trajectory.

To mitigate this problem, MALA introduces a one-dimensional logistic chaotic sequence $z_{t}\subset \left(0,1\right)$ to perturb the energy factor. Specifically, it first randomly initializes a seed $z_{0}\in \left(0,1\right)$ outside the interval of extreme values to generate the chaotic sequence $z_{t}$, where $z_{t}$ is employed as the perturbation term at iteration *t*. The logistic map is defined as

(4) $$z_{t}=\mu \;z_{t-1}\left(1-z_{t-1}\right),\;t=1,2,\ldots ,T$$

where the parameter μ is fixed to the widely adopted standard value of 4 in the logistic map and not treated as a control parameter in the optimization process [24,25]. Under this setting, the map exhibits chaotic behavior with good ergodic coverage over (0,1) and is employed as a bounded additive perturbation to modulate the energy gate near the switching threshold.

Based on the original energy formulation of ALA in Equation (2), a bounded chaotic perturbation term is incorporated to introduce controlled temporal variability into the energy gate. In generation *t*, the energy of individual *i* is redefined as follows:

(5) $$E_{i}^{\left(t\right)}=2\;\ln\;\left(\frac{1}{\mathrm{rand}}\right)\theta \left(t\right)+\varepsilon_{1}\left(z_{t}-0.5\right),\;\varepsilon_{1}>0.$$

Here, $\varepsilon_{1}>0$ controls the perturbation amplitude (default $\varepsilon_{1}=0.1$). The term $\left(z_{t}-0.5\right)\in \left(-0.5,0.5\right)$ enables symmetric positive–negative perturbation, ensuring unbiased disturbance to the energy factor. Moreover, the generated perturbation is strictly bounded within $\left(-\varepsilon_{1}/2,\;\varepsilon_{1}/2\right)$, without introducing significant disruptions to the original energy schedule. Given that $z_{t}$ demonstrates continuous variation within a limited amplitude, the significant temporal fluctuations in $E_{i}^{\left(t\right)}$ indicate persistent dynamic behavior, and moreover, the relative magnitude of these critical effects shows increasingly noticeable influence during the mid- and late stages when θ(t) demonstrates a systematic decrease.

Accordingly, some individuals temporarily enter exploration branches. This transition results in intermittent long-distance migration behavior and digging patterns. In this way, LCEF maintains an overall search tendency while avoiding premature fixation on exploitation behavior. Even in the convergence phase, the algorithm retains the ability to escape local optima.

#### 2.2.2. Elite Anchor Smoothing (EAS)

To improve the use of population-level statistics, MALA introduces elite anchor smoothing at each iteration.

Consider the set of current fitness values ${\left\{f\left(x_{i}^{\left(t\right)}\right)\right\}}_{i=1}^{N}$ and denote its median as ${\mathrm{med}}^{\left(t\right)}$. Here, f(·) denotes the objective function to be minimized. The elite set is defined as follows:

(6) $$E^{\left(t\right)}=\left\{x_{j}^{\left(t\right)}\;|\;f\left(x_{j}^{\left(t\right)}\right)\leq {\mathrm{med}}^{\left(t\right)}\right\}.$$

The average of the elite set is defined as follows:

(7) $${\bar{x}}^{\left(t\right)}=\frac{1}{|E^{\left(t\right)}|}\sum_{x\in E^{\left(t\right)}}x.$$

The median-based elite set improves robustness, even in the presence of suboptimal values or outliers. Therefore, EAS does not perform global recombination for all individuals, but instead applies a single smoothing update only to the current global best individual.

Let $x_{\mathrm{best}}^{\left(t\right)}$ be the global best individual, defined as the solution with the minimum objective value at iteration *t*. The anchor point is constructed as follows:

(8) $$c^{\left(t\right)}=\frac{1}{2}\left(x_{\mathrm{best}}^{\left(t\right)}+{\bar{x}}^{\left(t\right)}\right).$$

Then, only the position of the current best individual is replaced by $x_{i^{*}}^{\left(t+1\right)}\leftarrow c^{\left(t\right)}$, while the other $x_{i}^{\left(t+1\right)}$ remains unchanged.

The median-based elite set is used to reduce sensitivity to extreme fitness values, which improves robustness compared to mean-based selection. By updating only the global best individual, EAS avoids disrupting population diversity while providing a smoothed guidance direction. The averaging factor 1/2 provides a conservative correction, preventing overly aggressive shifts toward the elite center.

#### 2.2.3. Selective Small-Scale Fixing (S3-F)

MALA adopts Selective Small-Scale Fixing (S3-F) as a refinement of ALA’s direct component-wise projection, aiming to alleviate constraint-violation clustering and late-stage feasibility oscillations.

The candidate position of individual *i* is denoted by $x_{i}^{\left(t+1\right)}$. For each dimension *j*, the violation indicator is defined as follows:

(9) $$v_{i,j}^{\left(t\right)}=\left\{\begin{array}{cc} 1, & x_{i,j}^{\left(t+1\right)}<lb_{j}\;\mathrm{or}\;x_{i,j}^{\left(t+1\right)}>ub_{j}\\ 0, & \mathrm{otherwise}. \end{array}\right.$$

The set of violated dimensions is defined as follows:

(10) $$V_{i}^{\left(t\right)}=\left\{j|v_{i,j}^{\left(t\right)}=1\right\}.$$

If $V_{i}^{\left(t\right)}\neq Ø$, the anchor point $c^{\left(t\right)}$ defined in Equation (8) is used as the reference to repair the violated dimensions:

(11) $$x_{i,j}^{\left(t+1\right)}\leftarrow c_{j}^{\left(t\right)}+\varepsilon_{2}\;\theta \left(t\right)\eta_{j}\;\left(ub_{j}-lb_{j}\right),\;j\in V_{i}^{\left(t\right)},\;\eta_{j}∼N\left(0,1\right).$$

Here, $\varepsilon_{2}>0$ controls the repair scale (default $\varepsilon_{2}=0.05$). The annealing factor θ(t) is inherited from the original ALA energy factors and is reused to ensure that the repair scale follows the same temporal decay pattern as the exploration or exploitation transition. By centering the repair around the adaptive anchor point $c^{\left(t\right)}$, the correction is biased toward a representative and feasible region of the population, instead of forcing violated components directly onto the boundary. Subsequently, component-wise projection in Equation (3) is applied to ensure feasibility.

In this way, S3-F selectively repairs only the dimensions that violate the constraints relative to the adaptive anchor point, rather than enforcing strict feasibility for all dimensions in each iteration. This strategy suppresses excessive oscillations at the boundaries and ensures the continuity of the search process. Furthermore, decoupling boundary repair from full-dimensional updates enhances convergence stability. The effectiveness of each mechanism will be validated in subsequent experiments.

### 2.3. Integrated Algorithm Flow

**Figure 1:**
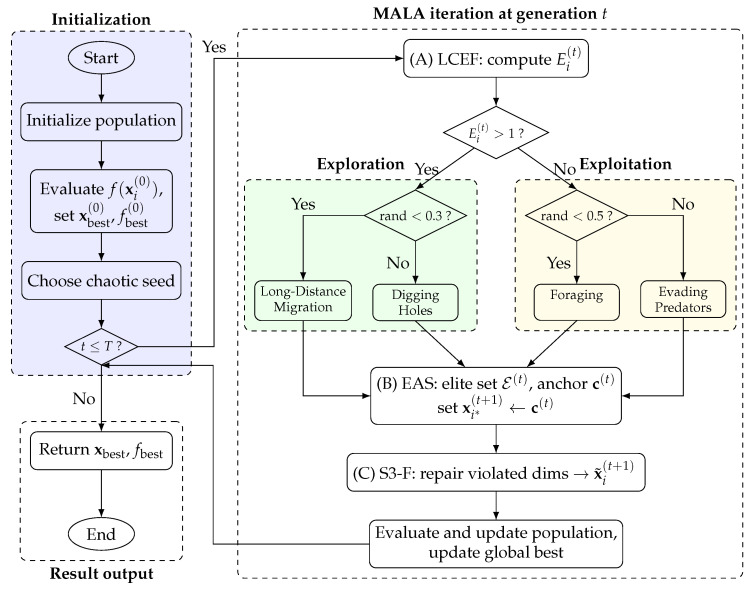
Flowchart of MALA.

The workflow of MALA is illustrated in Figure 1, and its pseudocode is described in Algorithm 1. At each iteration, the LCEF module first updates the logistic variable and individual energy coefficients. It then determines the behavioral branch of each individual based on the energy threshold and generates candidate solutions according to four behavioral actions. Subsequently, the EAS and S3-F modules are executed sequentially to perform elite anchor smoothing and selective small-scale boundary correction. Finally, a greedy selection strategy is used to update the individual solutions and the global best solution.   
**Algorithm 1:** Multi-Mechanism Artificial Lemming Algorithm (MALA).
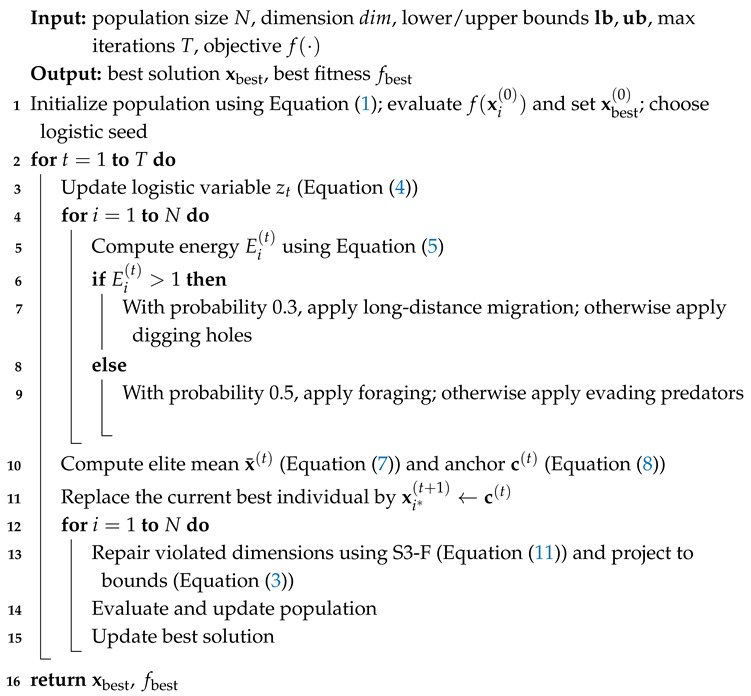


## 3. Experiments in the CEC2017 Benchmark Test Suite

The CEC2017 test suite is a widely used benchmark for single-objective continuous optimization and has been adopted in recent meta-heuristic studies [26,27,28]. It comprises 30 test functions. Following common practice, F2 was excluded and results were reported on the remaining 29 functions under 30-dimensional settings.

For the systematic validation of MALA’s effectiveness on general numerical optimization tasks, two experiments were conducted on CEC2017:

- Ablation Experiments: Under the same experimental settings, the main mechanisms of MALA are activated one by one to analyze the individual contributions and synergistic effects of each module.
- Comparative Experiments with Other Algorithms: Under a unified computational budget, MALA is compared with multiple classical and representative algorithms to evaluate its overall advantages across various function types.

### 3.1. Experimental Setup and Evaluation Metrics

All experiments followed the CEC2017 standardized protocol with dim=30 and population size N=30. Each function was run independently 30 times with T=500 iterations per run. The same set of seeds was used for all algorithms on each function to ensure fair stochastic comparisons.

All algorithms initialized their populations uniformly within the same search space ${\left[lb,ub\right]}^{dim}$.

For each function, the final fitness values from 30 independent runs were collected and summarized by the mean (Mean) and standard deviation (Std), with the best mean results highlighted in bold. Algorithms were ranked on each function according to Mean to obtain per-function ranks, and the average rank (AvgRank) over all 29 functions was reported.

### 3.2. Ablation Experiment

To analyze the independent contributions of each improved mechanism, an ablation study was performed with the original ALA as the baseline. This includes the following five variants:

- ALA: The original Artificial Lemming Algorithm;
- MALA_LCEF: Adds only LCEF to ALA, with other improvements disabled;
- MALA_EAS: Adds only EAS to ALA, with other improvements disabled;
- MALA_S3F: Adds only S3-F to ALA, with other improvements disabled;
- MALA: Multi-Mechanism Artificial Lemming Algorithm.

**Figure 2:**
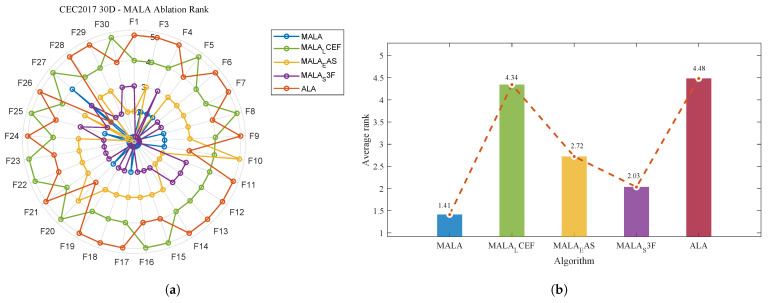
Ablation experimental results on the 30-dimensional CEC2017 test suite. (**a**) Ranking radar chart. (**b**) Average rank chart.

Figure 2a shows a Friedman ranking radar chart for the five variants on the 30-dimensional CEC2017 test suite. The MALA curve is generally closest to the center and ranks first on 19 out of 29 functions.

Figure 2b shows the average rank over the 29 functions. MALA achieves an AvgRank of 1.41, outperforming all single-module variants and the baseline ALA. Among the single-module variants, MALA_S3F (2.03) and MALA_EAS (2.72) outperform ALA in AvgRank. In contrast, MALA_LCEF (4.34) is close to ALA (4.48). However, when integrated with the other two mechanisms, it contributes to better overall performance in MALA.

Overall, MALA demonstrates consistent performance advantages over both its ablation variants and the original ALA across the evaluation metrics. Therefore, MALA is adopted in subsequent experiments and application studies.

### 3.3. Overall Comparison with Classical and Representative Algorithms

In this subsection, MALA is systematically compared with seven reference algorithms on the CEC2017 test suite, focusing on overall search capability, convergence efficiency, and result stability on complex functions. The comparison algorithms are selected to ensure relevance and fair coverage of different swarm intelligence paradigms. The closely related variants ALA and EALA [29] are included to verify the effectiveness of the proposed enhancement strategies. In addition, a wide range of swarm intelligence algorithms is considered, ranging from traditional swarm intelligence algorithms such as the Sine–Cosine Algorithm (SCA) [30] and Grey Wolf Optimizer (GWO) [31] to more recent and widely used approaches such as the Whale Optimization Algorithm (WOA) [32], Harris Hawks Optimization (HHO) [33], and Manta Ray Foraging Optimization (MRFO) [34].

MALA and ALA/EALA use the parameters recommended in the original studies. In contrast, SCA, GWO, WOA, HHO, and MRFO use typical parameter settings reported in their original publications. Where parameter ranges are suggested, the recommended default values are used.

#### 3.3.1. Comprehensive Results and Ranking Analysis

As shown in Table 1, MALA yields lower mean objective values for most of the tested functions, with its advantage particularly marked for mixed and composite functions, and achieves better expected solution quality under an identical evaluation budget. For instance, on the mixed function F22, the mean objective value obtained by MALA is 2.91 $\times \;10^{3}$, which is substantially lower than those of ALA (6.27$\;\times \;10^{3}$) and EALA (5.61 $\times \;10^{3}$), indicating superior solution quality under identical evaluation conditions.

Moreover, on composite functions such as F30, MALA exhibits a smaller standard deviation than ALA and EALA. The reduced performance dispersion across independent runs indicates enhanced robustness under constraints.

Table A1 presents the *p*-values obtained from the Wilcoxon rank-sum test between MALA and each comparison algorithm across the 29 test functions. For most functions, the difference between MALA and the comparison algorithms is statistically significant (p<0.05).

**Figure 3:**
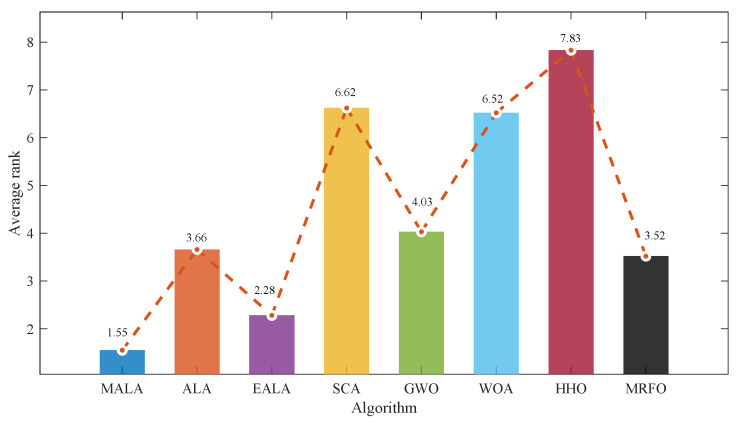
The average rank of 8 algorithms on the CEC2017 30-dimensional test suite.

Figure 3 shows the average ranking of the eight algorithms in the 30-dimensional CEC2017 test suite. MALA (1.55) achieves the lowest average rank overall. Thus, the proposed modifications provide a consistent advantage rather than isolated improvements on a few functions.

**Figure 4:**
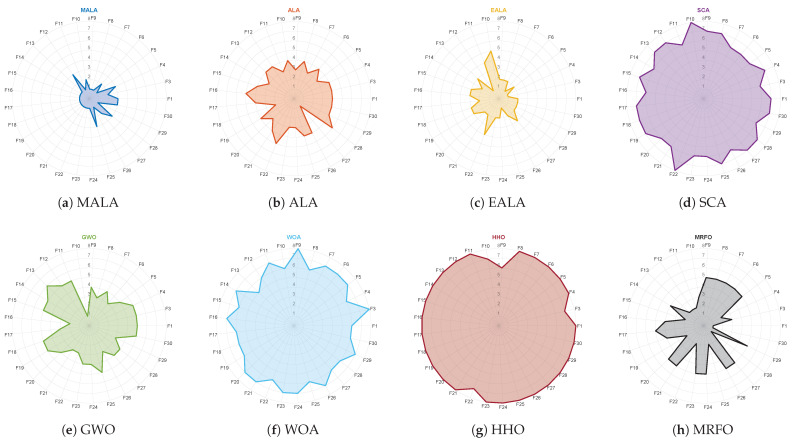
Radar chart of rankings for algorithms on the CEC2017 30-dimensional test suite.

Figure 4 shows that a smaller radius corresponds to a better rank for the corresponding function. This figure illustrates the ranking distribution across the 29 test functions. MALA achieves first place in 19 of them, and its curve is highly concentrated, indicating consistently stable performance across all functions. Furthermore, MALA remains competitive even on the few functions where it does not attain the best mean fitness.

#### 3.3.2. Convergence Behavior and Stability of MALA on Selected Functions

To analyze the convergence characteristics of each algorithm on different types of problems, this section selects six functions from the CEC2017 test suite. These functions cover basic multimodal functions (F5,F7,F9), mixed functions (F17), and composite functions (F21,F26).

**Figure 5:**
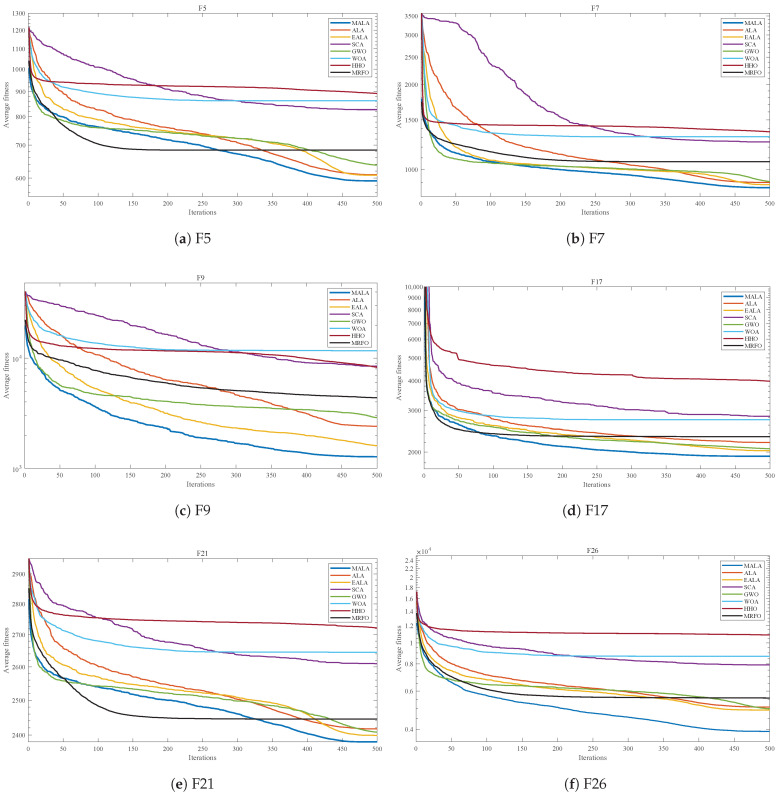
Average convergence curves for F5,F7,F9,F17,F21,F26 in the CEC2017 30-dimensional test suite.

Figure 5 shows the average convergence behavior of each algorithm on the selected CEC2017 functions, where the best-so-far objective value is recorded at every iteration in each run and the curves are averaged over 30 independent runs under the same population size and maximum iterations. For F5 and F21, MALA shows a convergence trend similar to several comparison algorithms in the early stage, while its objective value continues to decrease in later iterations. In contrast, the convergence curves of some algorithms show limited improvement in later iterations. Distinct differences in convergence behavior are observed among algorithms for F7, F9, F17, and F26, where MALA demonstrates a relatively stable convergence process.

**Figure 6:**
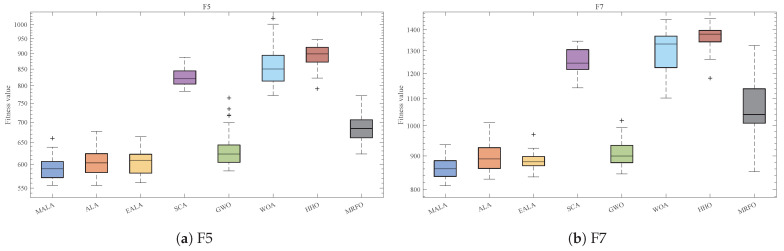

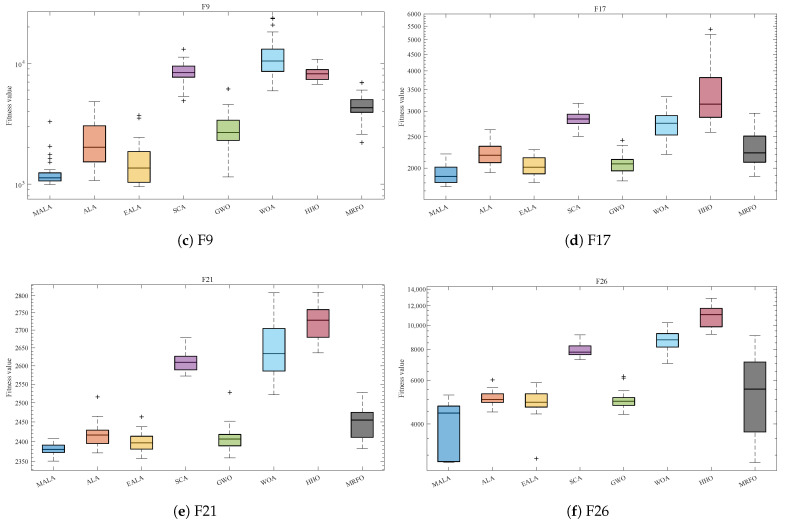
Final fitness box plots for F5,F7,F9,F17,F21,F26 in the CEC2017 30-dimensional test suite.

Moreover, the box plot in Figure 6 statistically characterizes the stability differences between algorithms across 30 independent runs. MALA presents a compact distribution across the six functions, with some competing algorithms exhibit longer boxes, indicating more outliers and a higher degree of uncertainty. This difference is particularly pronounced for hybrid and composite functions.

The average convergence curves and box plots demonstrate that MALA exhibits lower statistical dispersion and higher overall robustness than competing algorithms across all categories of the CEC2017 test suite.

### 3.4. Additional Benchmark Verification on CEC2014

To further examine the generalization performance of MALA, additional experiments were conducted on the CEC2014 benchmark suite [35]. The computational budget and experimental settings are consistent with those used for the CEC2017 test suite.

Table 2 presents the overall results for the CEC2014 test suite, including Mean±Std and average rank (AvgRank). The results show that MALA maintains competitive performance and achieves the lowest AvgRank (2.23), supporting the effectiveness of the proposed mechanisms on a different benchmark suite. The Wilcoxon rank-sum test results are provided in Table A2.

The above results indicate that MALA achieves overall performance better than ALA, EALA, and other representative swarm intelligence algorithms on the 30-dimensional CEC2017 and CEC2014 test suites. These findings further support the applicability to multi-threshold image segmentation.

## 4. Application in Color Multi-Threshold Image Segmentation

In this section, MALA is applied to color multi-threshold image segmentation. The stability and overall performance of the proposed algorithm are further examined in image segmentation tasks with increasing decision dimensionality.

**Figure 7:**
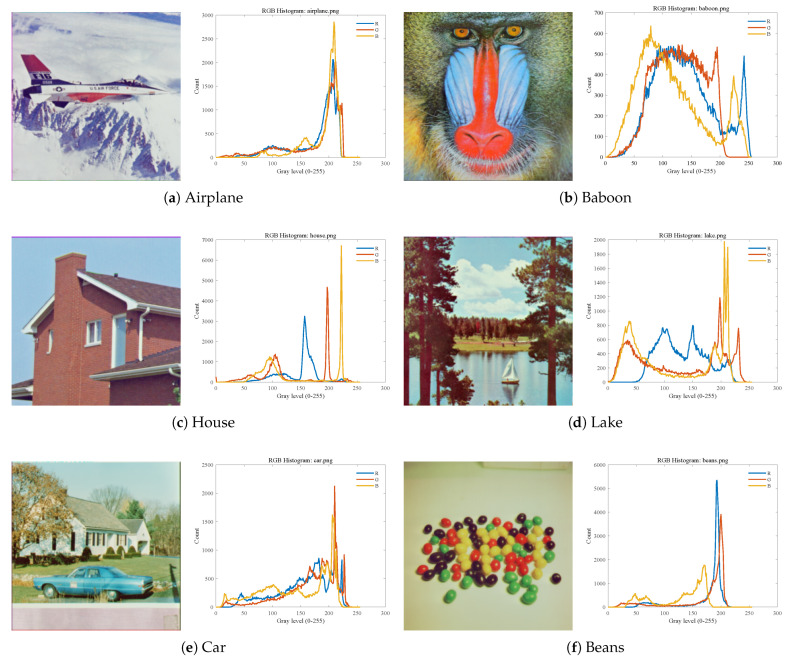
Test images and their RGB histograms.

Six color images were selected as the test set from the USC-SIPI image database (https://sipi.usc.edu/database/database.php?volume=misc, accessed on 1 January 2026). The selected images include Airplane, Baboon, House, Lake, Car, andBeans. The test images and their RGB histograms are shown in Figure 7.

### 4.1. Experimental Setup and Evaluation Metrics

Commonly used objective functions in multi-threshold segmentation include Otsu’s criterion [36] and Kapur’s entropy [37]. Otsu’s criterion is adopted due to its simplicity and ease of implementation [38,39], aiming to assess the stability and convergence behavior of optimizers under increasing decision dimensionality. The objective is defined as

(12) $$\max_{t}\;\mathrm{OBJ}\left(t\right)=\sum_{k=1}^{m+1}w_{k}{\left(\mu_{k}-\mu_{T}\right)}^{2}.$$

Here, the threshold vector for each channel satisfies t the condition $1\leq t_{1}<\cdots <t_{m}\leq 254$. When t is given, $w_{k}$ is the probability weight of the *k*-th segmented region, $\mu_{k}$ is the average intensity value of that region, and $\mu_{T}$ represents the average intensity value of the entire channel. These values are calculated using the normalized histogram, where p(i) denotes the probability of gray level *i* and $\sum_{i}p\left(i\right)=1$.

For color images, a channel-wise segmentation strategy is adopted. For each of the three RGB channels, *m* thresholds will be considered. This results in a decision dimension of 3m. Then, the objective functions of the three channels are combined to form an overall objective function. That is, $\mathrm{OBJ}={\mathrm{OBJ}}_{R}+{\mathrm{OBJ}}_{G}+{\mathrm{OBJ}}_{B}$. Additionally, to evaluate the results more comprehensively, the following three indicators are calculated:

- The Peak Signal-to-Noise Ratio (PSNR) is an indicator that measures the pixel-level distortion between the segmentation result and the original image [40]. It is defined as follows:
  (13) $$\mathrm{PSNR}=10\log_{10}\frac{255^{2}}{\mathrm{MSE}}.$$
  Here, MSE represents the Mean Squared Error. The higher the PSNR, the smaller the pixel distortion.
- The Structural Similarity Index (SSIM) is an indicator used to measure the retention of structural information [41], such as contours and textures, and is defined as follows:
  (14) $$\mathrm{SSIM}\left(x,y\right)=\frac{\left(2\mu_{x}\mu_{y}+C_{1}\right)\left(2\sigma_{xy}+C_{2}\right)}{\left(\mu_{x}^{2}+\mu_{y}^{2}+C_{1}\right)\left(\sigma_{x}^{2}+\sigma_{y}^{2}+C_{2}\right)}.$$
  Here, $\mu_{x}$ and $\mu_{y}$ represent the mean values, $\sigma_{x}$ and $\sigma_{y}$ represent the standard deviations, $\sigma_{xy}$ represents the covariance, and $C_{1}$ and $C_{2}$ are stabilization constants. The higher the SSIM value, the greater the structural similarity.
- The Feature Similarity Index (FSIM) describes the feature-level similarities between the segmented image and the original image and measures the perceptual consistency at the visual feature level [42]. It is based on two core visual features: phase consistency (PC) and gradient magnitude (GM). Let *x* and *y* denote the original image and the segmented image, respectively. $S_{i}$ denotes the local feature similarity at pixel *i*, which is jointly determined by the phase consistency and gradient magnitude similarities. The FSIM index evaluates feature similarity at the pixel level, and its mathematical expression is
  (15) $$\mathrm{FSIM}\left(x,y\right)=\frac{\sum_{i}S_{i}\;\cdot \;\max\left({\mathrm{PC}}_{i}^{x},{\mathrm{PC}}_{i}^{y}\right)}{\sum_{i}\max\left({\mathrm{PC}}_{i}^{x},{\mathrm{PC}}_{i}^{y}\right)}.$$

During implementation, the key metrics PSNR and SSIM are directly calculated on color images, while FSIM is calculated by converting RGB color images to grayscale images to ensure consistency and reproducibility of the results.

In the experiment, the number of thresholds *m* was set to {2,3,4,5}. The corresponding decision dimension 3m thus took values in {6,9,12,15}. For each image and threshold number *m*, each algorithm was run independently 20 times. The population size and the maximum number of iterations were N=30 and MaxIter=100, respectively, and the total number of fitness evaluations was set to 3000. Eight swarm intelligence algorithms, consistent with those used in the CEC2017 experiments, were compared under the same objective function, threshold constraint repair strategy, and stopping criteria.

### 4.2. Experimental Results and Discussion

#### 4.2.1. OBJ: Statistical Results and Convergence Curves

**Figure 8:**
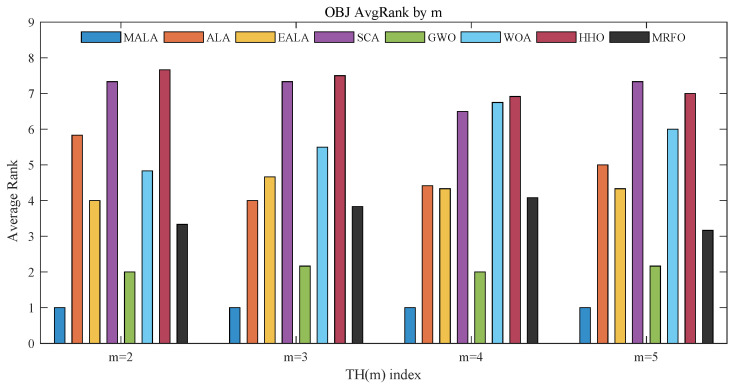
Average rankings based on OBJ.

Figure 8 shows that the average OBJ ranking varies with the number of thresholds *m*. Moreover, MALA attains the optimal value across all images and all threshold settings, demonstrating its considerable advantage in segmentation quality.

Table 3 presents the average objective function values (Mean±Std) for different threshold numbers *m*. Across different images and dimensions, MALA achieves the best objective values. For instance, in the Airplane image with m=5, MALA attains an objective value of $5.1056\times 10^{3}$, which is higher than those achieved by ALA ($5.0143\times 10^{3}$) and EALA ($5.0304\times 10^{3}$). Similarly, for the House image under m=3, MALA reaches $7.7975\times 10^{3}$, while the corresponding results of ALA and EALA are $7.6776\times 10^{3}$ and $7.6750\times 10^{3}$, respectively.

**Figure 9:**
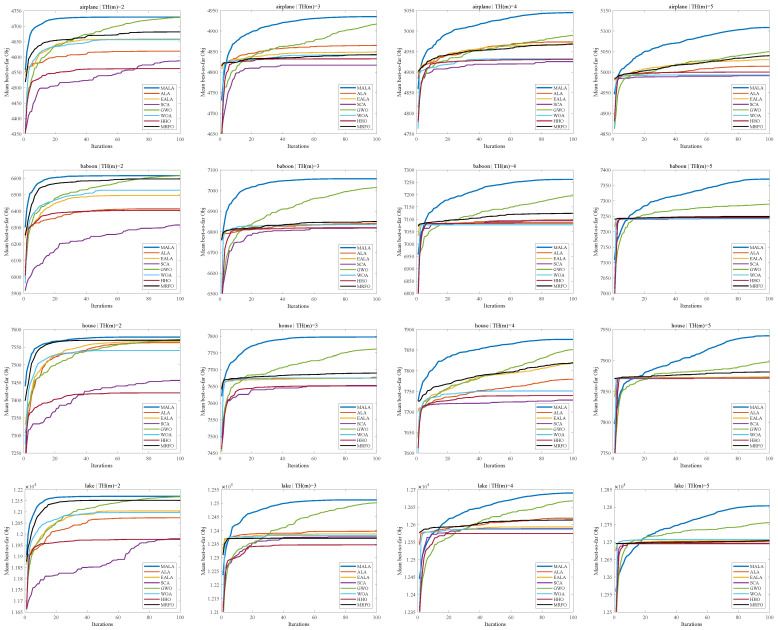

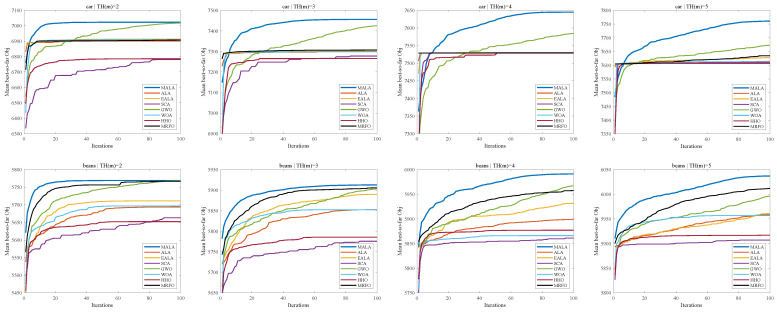
The average best-so-far OBJ convergence curves for six test images under different threshold numbers.

Furthermore, Figure 9 shows the average convergence curves of the best-so-far OBJ for six test images under m∈{2,3,4,5}. Across different images and threshold numbers, MALA quickly achieves high objective values in the early stages of iteration, showing a continuous upward trend without significant fluctuations. As *m* increases, the search space and problem complexity grow, leading to more pronounced performance differences among algorithms, which reflects the robustness of MALA in multi-threshold image segmentation tasks.

#### 4.2.2. PSNR, SSIM, FSIM: Ranking Trends and Statistical Results

**Figure 10:**
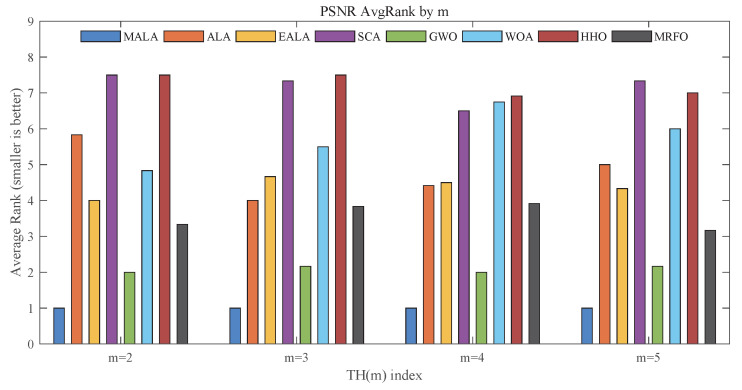
Average rankings based on PSNR.

Figure 10 shows the changes in the average PSNR ranking as the threshold number *m* increases. MALA consistently maintains the top ranking across all values of *m*, indicating relatively low pixel distortion in the segmented results.

This trend is consistent with the statistical results reported in Table A3. For instance, on the Airplane image with m=5, MALA achieves a PSNR value of $3.115\times 10^{1}$, which is higher than that of the second-ranked GWO ($2.974\times 10^{1}$), with a smaller standard deviation. GWO ranks second overall, while MRFO consistently holds the third position. In contrast, SCA and HHO occupy lower rankings for most *m* values, suggesting larger pixel-level distortion.

**Figure 11:**
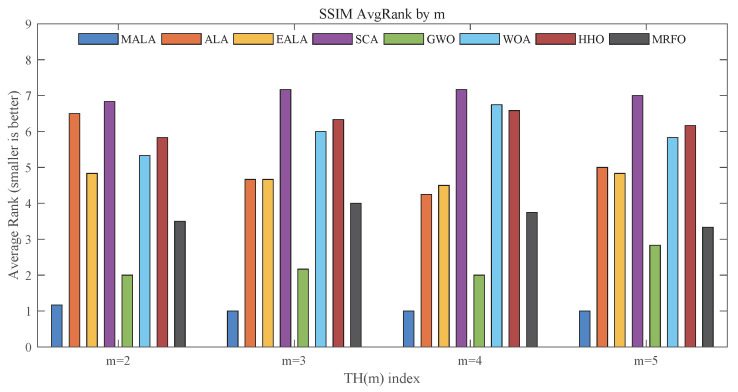
Average rankings based on SSIM.

Figure 11 compares the average SSIM rankings. Moreover, MALA consistently maintains the top ranking with minimal fluctuation as *m* increases, suggesting stable ability to preserve image structural information. However, GWO ranks second overall, while SCA occupies the last position, indicating weaker preservation of structural and textural information. These patterns appear consistent, and the observation is further supported by the quantitative results summarized in Table A4. On the Car image with m=3, MALA achieves an SSIM value of $8.623\times 10^{-1}$, which is higher than those obtained by ALA ($8.194\times 10^{-1}$) and EALA ($8.196\times 10^{-1}$).

**Figure 12:**
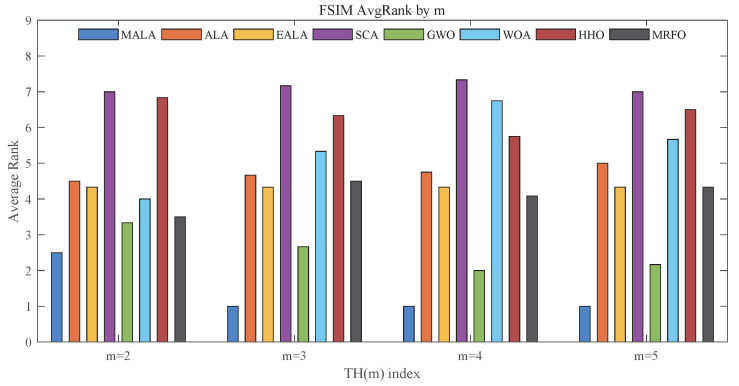
Average rankings based on FSIM.

As shown in Figure 12, the overall trend of FSIM rankings is consistent with the observed trends of PSNR and SSIM. MALA achieves the top ranking for each value of *m*. The results confirm that MALA effectively preserves perceptually relevant features, such as local contrast and salient structural cues, in multi-threshold segmentation.Thus, the detailed FSIM statistics in Table A5 support this trend.

### 4.3. Additional Application Verification on Remote Sensing Images

**Figure 13:**
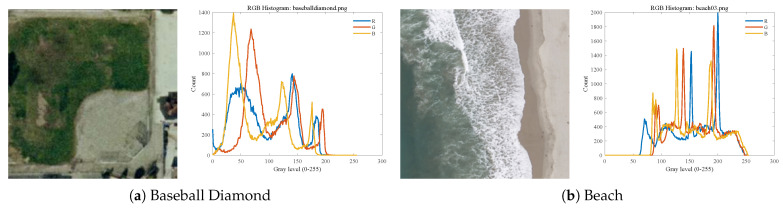
Remote sensing images and their RGB histograms.

To further validate the applicability and extensibility of MALA in practical scenarios, two remote sensing color images, Baseball Diamondand Beach, are adopted from the UCM Dataset for additional verification in remote sensing image segmentation tasks. Remote sensing images contain multiple land-cover types, such as vegetation, roads, and water, with distinct color distributions, making these data particularly suitable for color multi-threshold segmentation [43]. Furthermore, the selected remote sensing images and their corresponding RGB histograms exhibit characteristics consistent with those illustrated in Figure 13. The experimental settings and evaluation metrics are consistent with those used in previous sections.

**Figure 14:**
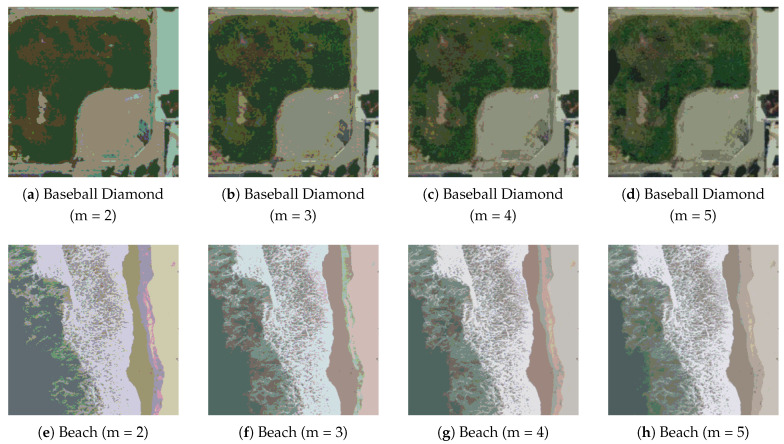
Segmentation results of remote sensing images under different threshold numbers.

Figure 14 shows the segmentation results under different threshold numbers. As the number of thresholds increases, the main land-cover regions are further subdivided, the color layers become richer, and the region boundaries remain clear.

For these remote sensing images, the OBJ statistics shown in Table 4 indicate that MALA is still able to obtain competitive objective function values under different threshold settings, with relatively small standard deviations, indicating stable overall performance. In addition, the detailed results of PSNR, SSIM, and FSIM are listed in Table A6, Table A7 and Table A8. MALA achieves the lowest average ranking across these metrics. This suggests that the improvement in objective value is achieved without sacrificing structural preservation or visual similarity.

In summary, MALA also exhibits good adaptability and stable performance in remote sensing color multi-threshold segmentation tasks.

## 5. Discussion and Conclusions

In this paper, MALA is designed to enhance the stability and adaptability of ALA, particularly for color multi-threshold image segmentation problems where the search space expands as the number of thresholds increases. The effectiveness of MALA has been validated through a series of numerical experiments. From the algorithm design perspective, MALA improves the convergence behavior and constraint-handling capability of ALA in complex search spaces by introducing complementary stabilization mechanisms, without altering the original behavioral structure of ALA.

Based on the results of the numerical experiments, MALA demonstrates relatively smoother convergence behavior on various functions in the CEC2017 test suite compared to the original ALA and several representative algorithms. Moreover, the average fitness values and average rankings are improved in most cases, with reduced performance variability across different function categories. In the application of color multi-threshold image segmentation, MALA is able to obtain higher Otsu objective values under a limited computational budget, even as the number of thresholds and the dimensionality of the search space increase. MALA performs well in PSNR, SSIM, and FSIM, and the results remain stable across different settings. These results show that combining the three mechanisms improves the robustness of MALA in constrained optimization problems.

This study has some limitations that warrant additional investigation. Moreover, the MALA approach still demonstrates reliance on a limited number of manually defined control parameters, and furthermore, more systematic analysis is necessary to evaluate their generalization across different segmentation scenarios. Given that the experiments on multi-threshold image segmentation are primarily based on traditional natural image datasets and the Otsu objective function, the experiments have not been extended to more complex application scenarios. Nevertheless, such scenarios involve stronger noise interference and more complex semantic structures.

To address these shortcomings, future research may explore several directions. Firstly, it would be meaningful to evaluate the applicability of MALA to other image analysis tasks such as medical image segmentation and multi-objective image optimization. Another direction is to combine MALA with other optimization strategies, which may improve its performance.

Overall, MALA extends the original ALA framework and shows suitability for color multi-threshold image segmentation.

## Figures and Tables

**Table 1:** Mean and Std of 30-dimensional CEC2017 test suite.

Func.	Type	MALA	ALA	EALA	SCA	GWO	WOA	HHO	MRFO
F1	Mean	5.37 × 10^4^	3.73 × 10^6^	8.10 × 10^3^	2.15 × 10^10^	2.72 × 10^9^	4.65 × 10^9^	4.95 × 10^10^	**3.88 × 10^3^**
	Std	5.89 × 10^4^	3.62 × 10^6^	7.30 × 10^3^	3.10 × 10^9^	2.21 × 10^9^	1.15 × 10^9^	8.24 × 10^9^	4.71 × 10^3^
F3	Mean	1.81 × 10^4^	2.85 × 10^4^	**1.04 × 10^4^**	8.75 × 10^4^	6.54 × 10^4^	2.61 × 10^5^	9.07 × 10^4^	2.05 × 10^4^
	Std	6.04 × 10^3^	9.44 × 10^3^	5.61 × 10^3^	2.06 × 10^4^	1.20 × 10^4^	8.49 × 10^4^	3.20 × 10^3^	5.08 × 10^3^
F4	Mean	5.12 × 10^2^	5.21 × 10^2^	**4.90 × 10^2^**	3.02 × 10^3^	6.77 × 10^2^	1.27 × 10^3^	1.30 × 10^4^	4.93 × 10^2^
	Std	2.54 × 10^1^	2.10 × 10^1^	2.11 × 10^1^	9.11 × 10^2^	1.96 × 10^2^	2.90 × 10^2^	3.33 × 10^3^	2.21 × 10^1^
F5	Mean	**5.92 × 10^2^**	6.09 × 10^2^	6.08 × 10^2^	8.27 × 10^2^	6.37 × 10^2^	8.62 × 10^2^	8.92 × 10^2^	6.84 × 10^2^
	Std	2.64 × 10^1^	3.18 × 10^1^	2.79 × 10^1^	3.09 × 10^1^	4.50 × 10^1^	6.50 × 10^1^	3.75 × 10^1^	3.57 × 10^1^
F6	Mean	6.06 × 10^2^	6.13 × 10^2^	**6.04 × 10^2^**	6.65 × 10^2^	6.13 × 10^2^	6.79 × 10^2^	6.83 × 10^2^	6.35 × 10^2^
	Std	3.96 × 10^0^	4.82 × 10^0^	2.18 × 10^0^	5.74 × 10^0^	4.76 × 10^0^	1.42 × 10^1^	8.10 × 10^0^	1.42 × 10^1^
F7	Mean	**8.62 × 10^2^**	8.99 × 10^2^	8.84 × 10^2^	1.25 × 10^3^	9.07 × 10^2^	1.31 × 10^3^	1.36 × 10^3^	1.07 × 10^3^
	Std	3.08 × 10^1^	4.61 × 10^1^	2.66 × 10^1^	5.16 × 10^1^	4.19 × 10^1^	9.26 × 10^1^	5.67 × 10^1^	1.05 × 10^2^
F8	Mean	**8.88 × 10^2^**	9.08 × 10^2^	9.02 × 10^2^	1.10 × 10^3^	9.04 × 10^2^	1.08 × 10^3^	1.12 × 10^3^	9.59 × 10^2^
	Std	2.20 × 10^1^	3.37 × 10^1^	2.77 × 10^1^	2.20 × 10^1^	2.94 × 10^1^	6.58 × 10^1^	2.48 × 10^1^	2.73 × 10^1^
F9	Mean	**1.28 × 10^3^**	2.40 × 10^3^	1.61 × 10^3^	8.46 × 10^3^	2.88 × 10^3^	1.17 × 10^4^	8.26 × 10^3^	4.37 × 10^3^
	Std	4.50 × 10^2^	1.11 × 10^3^	7.12 × 10^2^	1.66 × 10^3^	1.02 × 10^3^	4.77 × 10^3^	1.10 × 10^3^	1.01 × 10^3^
F10	Mean	5.39 × 10^3^	5.92 × 10^3^	6.38 × 10^3^	8.89 × 10^3^	**5.17 × 10^3^**	7.58 × 10^3^	8.27 × 10^3^	5.58 × 10^3^
	Std	8.71 × 10^2^	8.96 × 10^2^	7.77 × 10^2^	3.41 × 10^2^	1.70 × 10^3^	4.98 × 10^2^	5.55 × 10^2^	7.32 × 10^2^
F11	Mean	**1.22 × 10^3^**	1.27 × 10^3^	1.29 × 10^3^	4.16 × 10^3^	2.34 × 10^3^	8.92 × 10^3^	1.20 × 10^4^	1.26 × 10^3^
	Std	3.28 × 10^1^	4.46 × 10^1^	8.78 × 10^1^	1.13 × 10^3^	1.05 × 10^3^	5.17 × 10^3^	2.65 × 10^3^	4.79 × 10^1^
F12	Mean	1.07 × 10^6^	2.40 × 10^6^	**3.21 × 10^5^**	2.69 × 10^9^	8.89 × 10^7^	4.82 × 10^8^	1.01 × 10^10^	5.65 × 10^5^
	Std	5.21 × 10^5^	1.83 × 10^6^	2.55 × 10^5^	8.25 × 10^8^	9.64 × 10^7^	3.36 × 10^8^	3.48 × 10^9^	5.80 × 10^5^
F13	Mean	**1.47 × 10^4^**	4.67 × 10^4^	2.54 × 10^4^	1.12 × 10^9^	2.32 × 10^7^	1.44 × 10^7^	3.32 × 10^9^	2.50 × 10^4^
	Std	1.00 × 10^4^	3.07 × 10^4^	2.44 × 10^4^	4.52 × 10^8^	4.92 × 10^7^	1.27 × 10^7^	3.29 × 10^9^	2.06 × 10^4^
F14	Mean	**1.68 × 10^3^**	1.89 × 10^3^	1.73 × 10^3^	9.04 × 10^5^	3.19 × 10^5^	2.49 × 10^6^	4.43 × 10^6^	2.37 × 10^4^
	Std	7.75 × 10^1^	2.49 × 10^2^	2.29 × 10^2^	6.11 × 10^5^	4.42 × 10^5^	2.78 × 10^6^	4.71 × 10^6^	2.34 × 10^4^
F15	Mean	**5.00 × 10^3^**	2.14 × 10^4^	1.51 × 10^4^	6.57 × 10^7^	1.41 × 10^6^	1.11 × 10^7^	4.57 × 10^8^	9.82 × 10^3^
	Std	3.68 × 10^3^	1.18 × 10^4^	1.40 × 10^4^	5.20 × 10^7^	2.11 × 10^6^	1.80 × 10^7^	6.56 × 10^8^	1.00 × 10^4^
F16	Mean	**2.43 × 10^3^**	2.71 × 10^3^	2.68 × 10^3^	4.10 × 10^3^	2.50 × 10^3^	4.49 × 10^3^	5.13 × 10^3^	2.69 × 10^3^
	Std	2.52 × 10^2^	3.49 × 10^2^	3.32 × 10^2^	2.58 × 10^2^	2.84 × 10^2^	7.13 × 10^2^	6.05 × 10^2^	4.05 × 10^2^
F17	Mean	**1.92 × 10^3^**	2.20 × 10^3^	2.03 × 10^3^	2.83 × 10^3^	2.07 × 10^3^	2.74 × 10^3^	3.98 × 10^3^	2.33 × 10^3^
	Std	1.41 × 10^2^	1.76 × 10^2^	1.45 × 10^2^	1.68 × 10^2^	1.60 × 10^2^	2.45 × 10^2^	3.28 × 10^3^	3.05 × 10^2^
F18	Mean	**4.07 × 10^4^**	7.37 × 10^4^	7.47 × 10^4^	1.60 × 10^7^	1.70 × 10^6^	1.35 × 10^7^	5.80 × 10^7^	2.23 × 10^5^
	Std	2.28 × 10^4^	3.57 × 10^4^	6.00 × 10^4^	8.86 × 10^6^	1.92 × 10^6^	1.52 × 10^7^	6.57 × 10^7^	1.41 × 10^5^
F19	Mean	**7.44 × 10^3^**	2.20 × 10^4^	9.96 × 10^3^	1.04 × 10^8^	2.05 × 10^6^	2.06 × 10^7^	5.55 × 10^8^	9.85 × 10^3^
	Std	4.53 × 10^3^	2.11 × 10^4^	8.81 × 10^3^	5.35 × 10^7^	6.68 × 10^6^	1.81 × 10^7^	5.37 × 10^8^	9.06 × 10^3^
F20	Mean	**2.31 × 10^3^**	2.49 × 10^3^	2.41 × 10^3^	2.94 × 10^3^	2.53 × 10^3^	2.94 × 10^3^	2.99 × 10^3^	2.67 × 10^3^
	Std	1.13 × 10^2^	1.77 × 10^2^	1.83 × 10^2^	1.61 × 10^2^	2.10 × 10^2^	2.37 × 10^2^	1.86 × 10^2^	2.46 × 10^2^
F21	Mean	**2.38 × 10^3^**	2.42 × 10^3^	2.40 × 10^3^	2.61 × 10^3^	2.41 × 10^3^	2.65 × 10^3^	2.72 × 10^3^	2.45 × 10^3^
	Std	1.41 × 10^1^	3.17 × 10^1^	2.44 × 10^1^	2.73 × 10^1^	3.05 × 10^1^	6.99 × 10^1^	4.84 × 10^1^	3.98 × 10^1^
F22	Mean	**2.91 × 10^3^**	6.27 × 10^3^	5.61 × 10^3^	9.85 × 10^3^	5.51 × 10^3^	8.12 × 10^3^	9.32 × 10^3^	4.23 × 10^3^
	Std	1.43 × 10^3^	2.17 × 10^3^	2.81 × 10^3^	1.47 × 10^3^	1.89 × 10^3^	1.77 × 10^3^	8.70 × 10^2^	2.68 × 10^3^
F23	Mean	**2.74 × 10^3^**	2.77 × 10^3^	2.76 × 10^3^	3.08 × 10^3^	2.79 × 10^3^	3.15 × 10^3^	3.50 × 10^3^	2.86 × 10^3^
	Std	2.75 × 10^1^	3.31 × 10^1^	3.43 × 10^1^	4.93 × 10^1^	4.95 × 10^1^	1.17 × 10^2^	1.64 × 10^2^	7.38 × 10^1^
F24	Mean	**2.89 × 10^3^**	2.95 × 10^3^	2.93 × 10^3^	3.25 × 10^3^	2.97 × 10^3^	3.26 × 10^3^	3.69 × 10^3^	3.05 × 10^3^
	Std	2.27 × 10^1^	3.73 × 10^1^	3.66 × 10^1^	4.00 × 10^1^	6.56 × 10^1^	8.81 × 10^1^	1.75 × 10^2^	1.05 × 10^2^
F25	Mean	2.91 × 10^3^	2.91 × 10^3^	**2.90 × 10^3^**	3.57 × 10^3^	3.06 × 10^3^	3.23 × 10^3^	4.81 × 10^3^	2.90 × 10^3^
	Std	1.97 × 10^1^	2.00 × 10^1^	1.73 × 10^1^	1.90 × 10^2^	8.46 × 10^1^	8.22 × 10^1^	6.29 × 10^2^	1.71 × 10^1^
F26	Mean	**3.91 × 10^3^**	5.07 × 10^3^	4.91 × 10^3^	7.92 × 10^3^	4.97 × 10^3^	8.67 × 10^3^	1.09 × 10^4^	5.57 × 10^3^
	Std	9.67 × 10^2^	3.56 × 10^2^	5.15 × 10^2^	4.21 × 10^2^	4.11 × 10^2^	8.44 × 10^2^	1.12 × 10^3^	1.92 × 10^3^
F27	Mean	3.24 × 10^3^	**3.23 × 10^3^**	3.25 × 10^3^	3.55 × 10^3^	3.27 × 10^3^	3.54 × 10^3^	4.16 × 10^3^	3.30 × 10^3^
	Std	1.77 × 10^1^	1.66 × 10^1^	3.13 × 10^1^	6.52 × 10^1^	3.24 × 10^1^	1.65 × 10^2^	3.29 × 10^2^	7.57 × 10^1^
F28	Mean	3.26 × 10^3^	3.52 × 10^3^	3.23 × 10^3^	4.43 × 10^3^	3.47 × 10^3^	3.88 × 10^3^	6.61 × 10^3^	**3.23 × 10^3^**
	Std	2.43 × 10^1^	7.64 × 10^2^	3.29 × 10^1^	3.40 × 10^2^	1.03 × 10^2^	2.43 × 10^2^	7.73 × 10^2^	2.31 × 10^1^
F29	Mean	**3.69 × 10^3^**	4.04 × 10^3^	3.90 × 10^3^	5.30 × 10^3^	4.02 × 10^3^	5.53 × 10^3^	7.43 × 10^3^	4.08 × 10^3^
	Std	1.51 × 10^2^	2.01 × 10^2^	2.48 × 10^2^	3.44 × 10^2^	1.74 × 10^2^	5.54 × 10^2^	1.13 × 10^3^	3.24 × 10^2^
F30	Mean	2.86 × 10^4^	6.69 × 10^4^	1.51 × 10^4^	1.92 × 10^8^	1.28 × 10^7^	8.57 × 10^7^	7.79 × 10^8^	**1.46 × 10^4^**
	Std	1.44 × 10^4^	8.87 × 10^4^	6.08 × 10^3^	9.21 × 10^7^	1.28 × 10^7^	7.43 × 10^7^	5.20 × 10^8^	6.30 × 10^3^

**Table 2:** Mean, Std and AvgRank of 30-dimensional CEC2014 test suite.

Func.	Type	MALA	ALA	EALA	SCA	GWO	WOA	HHO	MRFO
F1	Mean	1.16 × 10^7^	2.26 × 10^7^	**3.39 × 10^6^**	5.40 × 10^8^	9.84 × 10^7^	2.40 × 10^8^	9.40 × 10^8^	5.50 × 10^6^
	Std	6.79 × 10^6^	1.17 × 10^7^	2.46 × 10^6^	1.78 × 10^8^	5.99 × 10^7^	8.09 × 10^7^	2.53 × 10^8^	3.28 × 10^6^
F2	Mean	6.56 × 10^4^	3.90 × 10^6^	1.36 × 10^4^	2.99 × 10^10^	3.29 × 10^9^	7.25 × 10^9^	6.75 × 10^10^	**1.19 × 10^4^**
	Std	6.01 × 10^4^	4.57 × 10^6^	1.19 × 10^4^	4.48 × 10^9^	2.23 × 10^9^	2.97 × 10^9^	9.43 × 10^9^	1.18 × 10^4^
F3	Mean	7.42 × 10^3^	1.52 × 10^4^	**6.56 × 10^3^**	8.31 × 10^4^	5.53 × 10^4^	1.26 × 10^5^	8.41 × 10^4^	1.26 × 10^4^
	Std	3.18 × 10^3^	8.21 × 10^3^	8.37 × 10^3^	1.81 × 10^4^	1.55 × 10^4^	6.49 × 10^4^	6.63 × 10^3^	1.15 × 10^4^
F4	Mean	5.20 × 10^2^	5.58 × 10^2^	5.24 × 10^2^	2.67 × 10^3^	6.97 × 10^2^	1.35 × 10^3^	1.17 × 10^4^	**4.99 × 10^2^**
	Std	3.60 × 10^1^	4.28 × 10^1^	3.73 × 10^1^	6.26 × 10^2^	9.25 × 10^1^	3.44 × 10^2^	3.20 × 10^3^	3.41 × 10^1^
F5	Mean	5.21 × 10^2^	5.21 × 10^2^	5.21 × 10^2^	5.21 × 10^2^	5.21 × 10^2^	5.21 × 10^2^	**5.21 × 10^2^**	5.21 × 10^2^
	Std	1.05 × 10^−1^	1.27 × 10^−1^	1.67 × 10^−1^	5.49 × 10^−2^	5.03 × 10^−2^	9.39 × 10^−2^	1.76 × 10^−1^	1.86 × 10^−1^
F6	Mean	**6.12 × 10^2^**	6.23 × 10^2^	6.16 × 10^2^	6.38 × 10^2^	6.17 × 10^2^	6.39 × 10^2^	6.42 × 10^2^	6.23 × 10^2^
	Std	2.43 × 10^0^	4.79 × 10^0^	3.27 × 10^0^	2.46 × 10^0^	3.32 × 10^0^	3.68 × 10^0^	2.51 × 10^0^	3.10 × 10^0^
F7	Mean	7.00 × 10^2^	7.01 × 10^2^	7.00 × 10^2^	9.53 × 10^2^	7.31 × 10^2^	7.48 × 10^2^	1.17 × 10^3^	**7.00 × 10^2^**
	Std	9.19 × 10^−2^	1.11 × 10^−1^	1.49 × 10^−2^	4.63 × 10^1^	2.02 × 10^1^	1.53 × 10^1^	1.03 × 10^2^	1.11 × 10^−2^
F8	Mean	**8.70 × 10^2^**	8.99 × 10^2^	8.78 × 10^2^	1.09 × 10^3^	8.98 × 10^2^	1.04 × 10^3^	1.10 × 10^3^	9.35 × 10^2^
	Std	1.81 × 10^1^	2.55 × 10^1^	2.03 × 10^1^	1.97 × 10^1^	2.53 × 10^1^	3.56 × 10^1^	2.33 × 10^1^	2.69 × 10^1^
F9	Mean	**9.94 × 10^2^**	1.00 × 10^3^	1.00 × 10^3^	1.22 × 10^3^	1.02 × 10^3^	1.20 × 10^3^	1.22 × 10^3^	1.07 × 10^3^
	Std	2.63 × 10^1^	3.16 × 10^1^	2.80 × 10^1^	2.31 × 10^1^	2.07 × 10^1^	5.15 × 10^1^	3.19 × 10^1^	2.83 × 10^1^
F10	Mean	**2.88 × 10^3^**	4.44 × 10^3^	4.58 × 10^3^	7.95 × 10^3^	4.04 × 10^3^	6.24 × 10^3^	7.53 × 10^3^	3.83 × 10^3^
	Std	6.31 × 10^2^	7.20 × 10^2^	8.71 × 10^2^	4.22 × 10^2^	1.27 × 10^3^	8.46 × 10^2^	5.99 × 10^2^	9.55 × 10^2^
F11	Mean	5.13 × 10^3^	6.19 × 10^3^	6.48 × 10^3^	8.84 × 10^3^	**4.64 × 10^3^**	7.72 × 10^3^	8.43 × 10^3^	4.97 × 10^3^
	Std	1.16 × 10^3^	8.23 × 10^2^	9.80 × 10^2^	3.73 × 10^2^	9.72 × 10^2^	7.68 × 10^2^	4.84 × 10^2^	5.80 × 10^2^
F12	Mean	**1.20 × 10^3^**	1.20 × 10^3^	1.20 × 10^3^	1.20 × 10^3^	1.20 × 10^3^	1.20 × 10^3^	1.20 × 10^3^	1.20 × 10^3^
	Std	5.64 × 10^−1^	6.61 × 10^−1^	7.94 × 10^−1^	4.48 × 10^−1^	9.71 × 10^−1^	6.96 × 10^−1^	5.43 × 10^−1^	8.38 × 10^−1^
F13	Mean	**1.30 × 10^3^**	1.30 × 10^3^	1.30 × 10^3^	1.30 × 10^3^	1.30 × 10^3^	1.30 × 10^3^	1.31 × 10^3^	1.30 × 10^3^
	Std	8.86 × 10^−2^	8.52 × 10^−2^	1.02 × 10^−1^	3.11 × 10^−1^	2.90 × 10^−1^	9.23 × 10^−1^	1.11 × 10^0^	1.25 × 10^−1^
F14	Mean	**1.40 × 10^3^**	1.40 × 10^3^	1.40 × 10^3^	1.49 × 10^3^	1.41 × 10^3^	1.42 × 10^3^	1.61 × 10^3^	1.40 × 10^3^
	Std	3.77 × 10^−2^	1.16 × 10^−1^	1.94 × 10^−1^	1.58 × 10^1^	8.96 × 10^0^	7.16 × 10^0^	3.13 × 10^1^	2.53 × 10^−1^
F15	Mean	**1.51 × 10^3^**	1.52 × 10^3^	1.52 × 10^3^	3.72 × 10^4^	1.80 × 10^3^	2.74 × 10^3^	9.87 × 10^4^	1.52 × 10^3^
	Std	3.37 × 10^0^	5.07 × 10^0^	5.31 × 10^0^	2.14 × 10^4^	7.04 × 10^2^	1.12 × 10^3^	4.61 × 10^4^	7.34 × 10^0^
F16	Mean	1.61 × 10^3^	1.61 × 10^3^	1.61 × 10^3^	1.61 × 10^3^	1.61 × 10^3^	1.61 × 10^3^	1.61 × 10^3^	1.61 × 10^3^
	Std	5.66 × 10^−1^	4.56 × 10^−1^	5.00 × 10^−1^	2.32 × 10^−1^	4.51 × 10^−1^	5.13 × 10^−1^	4.46 × 10^−1^	3.74 × 10^−1^
F17	Mean	**1.12 × 10^5^**	3.46 × 10^5^	2.27 × 10^5^	1.48 × 10^7^	3.78 × 10^6^	3.25 × 10^7^	8.09 × 10^7^	4.16 × 10^5^
	Std	8.27 × 10^4^	2.94 × 10^5^	2.13 × 10^5^	6.05 × 10^6^	3.62 × 10^6^	2.12 × 10^7^	5.31 × 10^7^	3.03 × 10^5^
F18	Mean	**2.46 × 10^3^**	1.66 × 10^4^	8.88 × 10^3^	4.76 × 10^8^	7.80 × 10^6^	8.60 × 10^6^	2.51 × 10^9^	7.10 × 10^3^
	Std	7.56 × 10^2^	9.53 × 10^3^	8.68 × 10^3^	2.90 × 10^8^	1.77 × 10^7^	8.26 × 10^6^	1.69 × 10^9^	6.68 × 10^3^
F19	Mean	**1.91 × 10^3^**	1.93 × 10^3^	1.93 × 10^3^	2.05 × 10^3^	1.96 × 10^3^	2.05 × 10^3^	2.26 × 10^3^	1.93 × 10^3^
	Std	1.46 × 10^0^	2.93 × 10^1^	2.84 × 10^1^	3.53 × 10^1^	3.84 × 10^1^	7.54 × 10^1^	1.02 × 10^2^	2.92 × 10^1^
F20	Mean	**2.85 × 10^3^**	3.80 × 10^3^	5.63 × 10^3^	5.45 × 10^4^	3.95 × 10^4^	1.66 × 10^5^	5.68 × 10^5^	2.32 × 10^4^
	Std	1.17 × 10^3^	1.15 × 10^3^	5.28 × 10^3^	3.45 × 10^4^	2.56 × 10^4^	1.25 × 10^5^	6.50 × 10^5^	1.44 × 10^4^
F21	Mean	**1.81 × 10^4^**	3.65 × 10^4^	3.80 × 10^4^	4.91 × 10^6^	2.02 × 10^6^	1.38 × 10^7^	3.67 × 10^7^	1.99 × 10^5^
	Std	8.03 × 10^3^	2.57 × 10^4^	3.25 × 10^4^	2.44 × 10^6^	2.75 × 10^6^	1.12 × 10^7^	2.63 × 10^7^	1.88 × 10^5^
F22	Mean	**2.54 × 10^3^**	2.60 × 10^3^	2.64 × 10^3^	3.36 × 10^3^	2.68 × 10^3^	3.31 × 10^3^	4.48 × 10^3^	2.87 × 10^3^
	Std	1.56 × 10^2^	1.59 × 10^2^	1.62 × 10^2^	1.81 × 10^2^	2.52 × 10^2^	3.31 × 10^2^	2.05 × 10^3^	3.02 × 10^2^
F23	Mean	2.54 × 10^3^	2.55 × 10^3^	2.51 × 10^3^	2.72 × 10^3^	2.65 × 10^3^	2.72 × 10^3^	**2.50 × 10^3^**	**2.50 × 10^3^**
	Std	2.75 × 10^1^	3.49 × 10^1^	2.20 × 10^1^	2.63 × 10^1^	1.37 × 10^1^	2.74 × 10^1^	0.00 × 10^0^	0.00 × 10^0^
F24	Mean	2.60 × 10^3^	2.60 × 10^3^	2.60 × 10^3^	2.63 × 10^3^	2.60 × 10^3^	2.61 × 10^3^	2.60 × 10^3^	**2.60 × 10^3^**
	Std	1.13 × 10^−1^	1.01 × 10^−1^	2.13 × 10^−2^	2.26 × 10^1^	3.65 × 10^−2^	4.99 × 10^0^	2.90 × 10^−4^	0.00 × 10^0^
F25	Mean	2.70 × 10^3^	2.70 × 10^3^	2.70 × 10^3^	2.74 × 10^3^	2.71 × 10^3^	2.72 × 10^3^	**2.70 × 10^3^**	**2.70 × 10^3^**
	Std	5.35 × 10^−4^	3.13 × 10^−2^	1.27 × 10^−3^	1.14 × 10^1^	4.72 × 10^0^	2.25 × 10^1^	0.00 × 10^0^	0.00 × 10^0^
F26	Mean	2.76 × 10^3^	**2.70 × 10^3^**	2.70 × 10^3^	2.70 × 10^3^	2.75 × 10^3^	2.73 × 10^3^	2.78 × 10^3^	2.70 × 10^3^
	Std	5.02 × 10^1^	1.07 × 10^−1^	1.82 × 10^1^	5.72 × 10^−1^	5.06 × 10^1^	6.44 × 10^1^	3.81 × 10^1^	1.46 × 10^−1^
F27	Mean	3.08 × 10^3^	3.22 × 10^3^	3.09 × 10^3^	3.86 × 10^3^	3.47 × 10^3^	3.97 × 10^3^	**2.90 × 10^3^**	**2.90 × 10^3^**
	Std	1.66 × 10^2^	3.16 × 10^2^	2.72 × 10^2^	2.55 × 10^2^	9.44 × 10^1^	2.55 × 10^2^	0.00 × 10^0^	0.00 × 10^0^
F28	Mean	3.32 × 10^3^	3.34 × 10^3^	3.26 × 10^3^	5.92 × 10^3^	4.19 × 10^3^	5.73 × 10^3^	**3.00 × 10^3^**	**3.00 × 10^3^**
	Std	2.69 × 10^2^	3.84 × 10^2^	5.38 × 10^2^	6.36 × 10^2^	3.55 × 10^2^	1.03 × 10^3^	0.00 × 10^0^	0.00 × 10^0^
F29	Mean	8.98 × 10^5^	1.00 × 10^7^	9.89 × 10^6^	4.09 × 10^7^	3.34 × 10^6^	1.82 × 10^7^	1.19 × 10^6^	**3.13 × 10^3^**
	Std	2.73 × 10^6^	4.29 × 10^6^	8.76 × 10^6^	1.76 × 10^7^	7.74 × 10^6^	1.27 × 10^7^	6.52 × 10^6^	1.55 × 10^2^
F30	Mean	**6.97 × 10^3^**	2.24 × 10^4^	1.21 × 10^4^	5.57 × 10^5^	9.64 × 10^4^	4.68 × 10^5^	4.84 × 10^5^	1.31 × 10^4^
	Std	1.98 × 10^3^	2.20 × 10^4^	1.32 × 10^4^	1.64 × 10^5^	5.67 × 10^4^	2.78 × 10^5^	1.05 × 10^6^	2.32 × 10^4^
AvgRank	–	**2.23**	3.63	3.03	7.07	4.77	6.23	6.20	2.83

**Table 3:** Mean and Std of OBJ on multi-threshold segmentation (higher is better).

Image	TH	Type	MALA	ALA	EALA	SCA	GWO	WOA	HHO	MRFO
Airplane	2	Mean	**4.7297 × 10^3^**	4.6189 × 10^3^	4.6582 × 10^3^	4.5875 × 10^3^	4.7286 × 10^3^	4.6565 × 10^3^	4.5622 × 10^3^	4.6815 × 10^3^
		Std	1.7624 × 10^−2^	7.9463 × 10^1^	8.4308 × 10^1^	9.4773 × 10^1^	4.0885 × 10^0^	7.1835 × 10^1^	9.1275 × 10^1^	7.8206 × 10^1^
	3	Mean	**4.9349 × 10^3^**	4.8649 × 10^3^	4.8488 × 10^3^	4.8171 × 10^3^	4.9168 × 10^3^	4.8416 × 10^3^	4.8324 × 10^3^	4.8425 × 10^3^
		Std	4.8624 × 10^−1^	3.1975 × 10^1^	3.3655 × 10^1^	1.9843 × 10^1^	2.0277 × 10^1^	3.2373 × 10^1^	2.8381 × 10^1^	4.3351 × 10^1^
	4	Mean	**5.0448 × 10^3^**	4.9734 × 10^3^	4.9692 × 10^3^	4.9257 × 10^3^	4.9892 × 10^3^	4.9323 × 10^3^	4.9312 × 10^3^	4.9682 × 10^3^
		Std	1.1952 × 10^0^	3.1180 × 10^1^	4.0577 × 10^1^	2.1069 × 10^1^	3.1415 × 10^1^	4.0911 × 10^1^	3.7974 × 10^1^	5.1373 × 10^1^
	5	Mean	**5.1086 × 10^3^**	5.0143 × 10^3^	5.0304 × 10^3^	4.9914 × 10^3^	5.0498 × 10^3^	4.9933 × 10^3^	5.0001 × 10^3^	5.0399 × 10^3^
		Std	2.0623 × 10^0^	2.6020 × 10^1^	2.2180 × 10^1^	9.7197 × 10^0^	2.1944 × 10^1^	1.7511 × 10^1^	2.3406 × 10^1^	3.9494 × 10^1^
Baboon	2	Mean	**6.6152 × 10^3^**	6.4145 × 10^3^	6.4952 × 10^3^	6.3155 × 10^3^	6.6150 × 10^3^	6.5273 × 10^3^	6.4051 × 10^3^	6.5957 × 10^3^
		Std	9.1797 × 10^−3^	1.7027 × 10^2^	1.4610 × 10^2^	1.7162 × 10^2^	1.4720 × 10^−1^	1.3508 × 10^2^	1.4626 × 10^2^	5.9921 × 10^1^
	3	Mean	**7.0577 × 10^3^**	6.8408 × 10^3^	6.8344 × 10^3^	6.8187 × 10^3^	7.0148 × 10^3^	6.8353 × 10^3^	6.8199 × 10^3^	6.8498 × 10^3^
		Std	5.5538 × 10^−1^	9.0046 × 10^1^	7.1528 × 10^1^	4.7167 × 10^1^	6.7020 × 10^1^	8.3079 × 10^1^	9.8734 × 10^1^	1.0874 × 10^2^
	4	Mean	**7.2619 × 10^3^**	7.0977 × 10^3^	7.0877 × 10^3^	7.0941 × 10^3^	7.1957 × 10^3^	7.0777 × 10^3^	7.0846 × 10^3^	7.1243 × 10^3^
		Std	1.0502 × 10^0^	4.0977 × 10^1^	3.0739 × 10^1^	2.2347 × 10^1^	4.9905 × 10^1^	2.7994 × 10^−12^	2.2376 × 10^1^	5.5708 × 10^1^
	5	Mean	**7.3704 × 10^3^**	7.2483 × 10^3^	7.2449 × 10^3^	7.2434 × 10^3^	7.2892 × 10^3^	7.2420 × 10^3^	7.2458 × 10^3^	7.2493 × 10^3^
		Std	2.0068 × 10^0^	2.0442 × 10^1^	2.1347 × 10^1^	8.4507 × 10^0^	1.6748 × 10^1^	8.2942 × 10^0^	1.7603 × 10^1^	2.0648 × 10^1^
House	2	Mean	**7.5786 × 10^3^**	7.5626 × 10^3^	7.5654 × 10^3^	7.4561 × 10^3^	7.5718 × 10^3^	7.5405 × 10^3^	7.4207 × 10^3^	7.5694 × 10^3^
		Std	3.8359 × 10^0^	2.3970 × 10^1^	3.3780 × 10^1^	6.3663 × 10^1^	2.1320 × 10^1^	4.1677 × 10^1^	1.0111 × 10^2^	1.7683 × 10^1^
	3	Mean	**7.7975 × 10^3^**	7.6776 × 10^3^	7.6750 × 10^3^	7.6530 × 10^3^	7.7612 × 10^3^	7.6753 × 10^3^	7.6512 × 10^3^	7.6899 × 10^3^
		Std	3.5424 × 10^−2^	2.6501 × 10^1^	3.1792 × 10^1^	2.5954 × 10^1^	3.3647 × 10^1^	3.7598 × 10^1^	4.7006 × 10^1^	5.4932 × 10^1^
	4	Mean	**7.8759 × 10^3^**	7.7796 × 10^3^	7.8179 × 10^3^	7.7290 × 10^3^	7.8513 × 10^3^	7.7510 × 10^3^	7.7398 × 10^3^	7.8195 × 10^3^
		Std	2.9689 × 10^0^	5.4415 × 10^1^	6.3239 × 10^1^	1.8557 × 10^1^	3.5995 × 10^1^	6.3735 × 10^1^	4.8611 × 10^1^	7.0405 × 10^1^
	5	Mean	**7.9398 × 10^3^**	7.8729 × 10^3^	7.8738 × 10^3^	7.8716 × 10^3^	7.8978 × 10^3^	7.8717 × 10^3^	7.8715 × 10^3^	7.8815 × 10^3^
		Std	2.2191 × 10^0^	1.0074 × 10^1^	1.0384 × 10^1^	2.4204 × 10^0^	2.4810 × 10^1^	4.5682 × 10^0^	3.9325 × 10^0^	1.8989 × 10^1^
Lake	2	Mean	**1.2170 × 10^4^**	1.2073 × 10^4^	1.2104 × 10^4^	1.1980 × 10^4^	1.2168 × 10^4^	1.2097 × 10^4^	1.1977 × 10^4^	1.2152 × 10^4^
		Std	7.1705 × 10^−3^	1.2648 × 10^2^	1.1121 × 10^2^	8.4810 × 10^1^	5.9337 × 10^0^	1.1231 × 10^2^	1.2964 × 10^2^	6.4755 × 10^1^
	3	Mean	**1.2512 × 10^4^**	1.2397 × 10^4^	1.2387 × 10^4^	1.2376 × 10^4^	1.2502 × 10^4^	1.2382 × 10^4^	1.2347 × 10^4^	1.2372 × 10^4^
		Std	2.0991 × 10^−1^	4.8930 × 10^1^	4.2247 × 10^1^	1.5130 × 10^1^	2.1804 × 10^1^	3.4212 × 10^1^	6.5031 × 10^1^	4.8430 × 10^0^
	4	Mean	**1.2690 × 10^4^**	1.2618 × 10^4^	1.2593 × 10^4^	1.2589 × 10^4^	1.2668 × 10^4^	1.2587 × 10^4^	1.2575 × 10^4^	1.2614 × 10^4^
		Std	6.8679 × 10^0^	5.2199 × 10^1^	4.1228 × 10^1^	2.5856 × 10^1^	2.4047 × 10^1^	2.6250 × 10^1^	3.3388 × 10^0^	5.0873 × 10^1^
	5	Mean	**1.2804 × 10^4^**	1.2701 × 10^4^	1.2704 × 10^4^	1.2696 × 10^4^	1.2756 × 10^4^	1.2708 × 10^4^	1.2695 × 10^4^	1.2704 × 10^4^
		Std	2.5680 × 10^0^	1.7462 × 10^1^	2.2401 × 10^1^	2.3122 × 10^0^	2.2506 × 10^1^	2.5556 × 10^1^	3.7325 × 10^−12^	1.8438 × 10^1^
Car	2	Mean	**7.0227 × 10^3^**	6.9012 × 10^3^	6.9128 × 10^3^	6.7810 × 10^3^	7.0178 × 10^3^	6.9094 × 10^3^	6.7855 × 10^3^	6.9074 × 10^3^
		Std	0.0000 × 10^0^	3.3254 × 10^1^	4.9620 × 10^1^	1.5428 × 10^2^	1.4626 × 10^1^	4.5821 × 10^1^	1.5378 × 10^2^	4.8248 × 10^1^
	3	Mean	**7.4559 × 10^3^**	7.3000 × 10^3^	7.3037 × 10^3^	7.2774 × 10^3^	7.4250 × 10^3^	7.2985 × 10^3^	7.2654 × 10^3^	7.3069 × 10^3^
		Std	3.2037 × 10^−1^	3.9003 × 10^1^	4.1457 × 10^1^	3.5421 × 10^1^	3.4957 × 10^1^	3.2765 × 10^1^	8.3505 × 10^1^	5.1260 × 10^1^
	4	Mean	**7.6449 × 10^3^**	7.5287 × 10^3^	7.5304 × 10^3^	7.5299 × 10^3^	7.5843 × 10^3^	7.5287 × 10^3^	7.5287 × 10^3^	7.5287 × 10^3^
		Std	2.0245 × 10^0^	2.7994 × 10^−12^	7.7361 × 10^0^	3.5251 × 10^0^	3.6522 × 10^1^	2.7994 × 10^−12^	2.7994 × 10^−12^	2.7994 × 10^−12^
	5	Mean	**7.7606 × 10^3^**	7.6139 × 10^3^	7.6278 × 10^3^	7.6091 × 10^3^	7.6731 × 10^3^	7.6129 × 10^3^	7.6070 × 10^3^	7.6348 × 10^3^
		Std	5.5164 × 10^0^	2.1343 × 10^1^	3.9334 × 10^1^	8.9475 × 10^0^	3.3479 × 10^1^	2.5361 × 10^1^	9.6170 × 10^0^	3.4948 × 10^1^
Beans	2	Mean	**5.7691 × 10^3^**	5.6938 × 10^3^	5.7111 × 10^3^	5.6633 × 10^3^	5.7685 × 10^3^	5.6968 × 10^3^	5.6521 × 10^3^	5.7667 × 10^3^
		Std	1.8662 × 10^−12^	8.8886 × 10^1^	8.8935 × 10^1^	6.6679 × 10^1^	2.0083 × 10^0^	8.1994 × 10^1^	6.0310 × 10^1^	1.0640 × 10^1^
	3	Mean	**5.9126 × 10^3^**	5.8525 × 10^3^	5.8898 × 10^3^	5.7755 × 10^3^	5.9029 × 10^3^	5.8520 × 10^3^	5.7855 × 10^3^	5.9057 × 10^3^
		Std	2.0409 × 10^0^	6.7737 × 10^1^	3.2725 × 10^1^	4.3507 × 10^1^	1.8875 × 10^1^	6.9498 × 10^1^	5.2016 × 10^1^	1.7999 × 10^1^
	4	Mean	**5.9912 × 10^3^**	5.8985 × 10^3^	5.9315 × 10^3^	5.8610 × 10^3^	5.9671 × 10^3^	5.8660 × 10^3^	5.8771 × 10^3^	5.9575 × 10^3^
		Std	1.5077 × 10^0^	4.3459 × 10^1^	6.0679 × 10^1^	1.7089 × 10^1^	2.9176 × 10^1^	4.1552 × 10^1^	3.2465 × 10^1^	5.0145 × 10^1^
	5	Mean	**6.0369 × 10^3^**	5.9584 × 10^3^	5.9611 × 10^3^	5.9070 × 10^3^	5.9961 × 10^3^	5.9568 × 10^3^	5.9168 × 10^3^	6.0118 × 10^3^
		Std	1.8353 × 10^0^	4.0180 × 10^1^	5.9369 × 10^1^	1.6331 × 10^1^	3.1287 × 10^1^	4.3183 × 10^1^	3.4219 × 10^1^	3.3022 × 10^1^
Rank			**1**	5	4	7	2	6	8	3

**Table 4:** Mean, Std, and AvgRank of OBJ for remote sensing images under multi-threshold segmentation.

Image	TH	Type	MALA	ALA	EALA	SCA	GWO	WOA	HHO	MRFO
Beach	2	Mean	**5.4450 × 10^3^**	5.4186 × 10^3^	5.4433 × 10^3^	5.2293 × 10^3^	5.4448 × 10^3^	5.3412 × 10^3^	5.2128 × 10^3^	5.4378 × 10^3^
		Std	9.3300 × 10^−13^	8.9750 × 10^1^	7.5576 × 10^0^	8.1553 × 10^1^	1.5854 × 10^−1^	1.2104 × 10^2^	1.2825 × 10^2^	3.2497 × 10^1^
	3	Mean	**5.7819 × 10^3^**	5.6542 × 10^3^	5.7463 × 10^3^	5.4444 × 10^3^	5.7650 × 10^3^	5.6504 × 10^3^	5.5045 × 10^3^	5.7528 × 10^3^
		Std	3.5451 × 10^0^	1.3756 × 10^2^	7.3701 × 10^1^	7.1209 × 10^1^	3.6420 × 10^1^	9.1860 × 10^1^	1.2053 × 10^2^	5.8930 × 10^1^
	4	Mean	**5.9568 × 10^3^**	5.7559 × 10^3^	5.7949 × 10^3^	5.6914 × 10^3^	5.8590 × 10^3^	5.7764 × 10^3^	5.6993 × 10^3^	5.8303 × 10^3^
		Std	5.9925 × 10^−1^	5.3293 × 10^1^	9.2177 × 10^1^	2.0534 × 10^1^	7.7347 × 10^1^	9.9350 × 10^1^	5.3066 × 10^1^	1.1144 × 10^2^
	5	Mean	**6.0212 × 10^3^**	5.8616 × 10^3^	5.8918 × 10^3^	5.8418 × 10^3^	5.9149 × 10^3^	5.8499 × 10^3^	5.8409 × 10^3^	5.8977 × 10^3^
		Std	5.1199 × 10^0^	4.5337 × 10^1^	4.8088 × 10^1^	1.6110 × 10^1^	2.8643 × 10^1^	3.2481 × 10^1^	2.9613 × 10^1^	3.7788 × 10^1^
Baseball Diamond	2	Mean	**5.7223 × 10^3^**	5.5909 × 10^3^	5.6492 × 10^3^	5.5646 × 10^3^	5.7204 × 10^3^	5.6705 × 10^3^	5.6086 × 10^3^	5.7093 × 10^3^
		Std	8.0443 × 10^−3^	8.5736 × 10^1^	9.6240 × 10^1^	7.8503 × 10^1^	8.4800 × 10^0^	6.8819 × 10^1^	7.6694 × 10^1^	4.6048 × 10^1^
	3	Mean	**6.0226 × 10^3^**	5.8547 × 10^3^	5.9329 × 10^3^	5.7619 × 10^3^	6.0134 × 10^3^	5.9163 × 10^3^	5.8506 × 10^3^	6.0098 × 10^3^
		Std	1.6824 × 10^−1^	1.4617 × 10^2^	1.5696 × 10^2^	8.2125 × 10^1^	2.0982 × 10^1^	1.0736 × 10^2^	7.8704 × 10^1^	5.1764 × 10^1^
	4	Mean	**6.1789 × 10^3^**	5.9846 × 10^3^	6.0002 × 10^3^	5.9947 × 10^3^	6.1498 × 10^3^	5.9991 × 10^3^	6.0097 × 10^3^	6.0541 × 10^3^
		Std	1.3778 × 10^0^	5.3447 × 10^0^	4.2200 × 10^1^	1.4240 × 10^1^	3.7781 × 10^1^	3.9348 × 10^1^	4.3065 × 10^1^	8.6617 × 10^1^
	5	Mean	**6.2607 × 10^3^**	6.1191 × 10^3^	6.1191 × 10^3^	6.1257 × 10^3^	6.2105 × 10^3^	6.1191 × 10^3^	6.1281 × 10^3^	6.1274 × 10^3^
		Std	3.2338 × 10^0^	1.8700 × 10^−12^	1.8700 × 10^−12^	1.3331 × 10^1^	3.6245 × 10^1^	1.8700 × 10^−12^	2.4758 × 10^1^	2.5433 × 10^1^
AvgRank			**1**	6.125	4.5	7.25	2	5.625	6.25	3.25

## Data Availability

All the data in this article can be obtained by contacting the corresponding author.

## Appendix A

**Table A1:** Wilcoxon rank-sum test *p*-values for MALA versus each comparison algorithm on the CEC2017 30D benchmark functions.

Func.	ALA	EALA	SCA	GWO	WOA	HHO	MRFO
F1	3.02 × 10^−11^	2.00 × 10^−6^	3.02 × 10^−11^	3.02 × 10^−11^	3.02 × 10^−11^	3.02 × 10^−11^	1.55 × 10^−9^
F3	8.29 × 10^−6^	8.29 × 10^−6^	3.02 × 10^−11^	3.02 × 10^−11^	3.02 × 10^−11^	3.02 × 10^−11^	1.91 × 10^−2^
F4	2.12 × 10^−1^	5.87 × 10^−4^	3.02 × 10^−11^	3.16 × 10^−10^	3.02 × 10^−11^	3.02 × 10^−11^	1.90 × 10^−3^
F5	4.51 × 10^−2^	2.71 × 10^−2^	3.02 × 10^−11^	5.09 × 10^−6^	3.02 × 10^−11^	3.02 × 10^−11^	9.92 × 10^−11^
F6	2.57 × 10^−7^	3.78 × 10^−2^	3.02 × 10^−11^	4.80 × 10^−7^	3.02 × 10^−11^	3.02 × 10^−11^	7.39 × 10^−11^
F7	2.00 × 10^−3^	3.20 × 10^−3^	3.02 × 10^−11^	2.60 × 10^−5^	3.02 × 10^−11^	3.02 × 10^−11^	2.87 × 10^−10^
F8	1.50 × 10^−2^	8.50 × 10^−2^	3.02 × 10^−11^	3.78 × 10^−2^	3.02 × 10^−11^	3.02 × 10^−11^	2.61 × 10^−10^
F9	2.03 × 10^−7^	5.01 × 10^−2^	3.02 × 10^−11^	1.69 × 10^−9^	3.02 × 10^−11^	3.02 × 10^−11^	4.50 × 10^−11^
F10	4.51 × 10^−2^	1.49 × 10^−4^	3.02 × 10^−11^	2.92 × 10^−2^	6.70 × 10^−11^	3.02 × 10^−11^	3.95 × 10^−1^
F11	1.39 × 10^−6^	7.70 × 10^−4^	3.02 × 10^−11^	3.02 × 10^−11^	3.02 × 10^−11^	3.02 × 10^−11^	7.20 × 10^−5^
F12	2.90 × 10^−3^	2.83 × 10^−8^	3.02 × 10^−11^	3.02 × 10^−11^	3.02 × 10^−11^	3.02 × 10^−11^	1.58 × 10^−4^
F13	1.43 × 10^−5^	2.12 × 10^−1^	3.02 × 10^−11^	3.02 × 10^−11^	3.02 × 10^−11^	3.02 × 10^−11^	7.01 × 10^−2^
F14	3.37 × 10^−5^	9.47 × 10^−1^	3.02 × 10^−11^	3.02 × 10^−11^	3.02 × 10^−11^	3.02 × 10^−11^	3.02 × 10^−11^
F15	5.46 × 10^−9^	2.50 × 10^−3^	3.02 × 10^−11^	4.08 × 10^−11^	3.02 × 10^−11^	3.02 × 10^−11^	1.76 × 10^−1^
F16	1.10 × 10^−3^	4.60 × 10^−3^	3.02 × 10^−11^	4.73 × 10^−1^	3.02 × 10^−11^	3.02 × 10^−11^	1.17 × 10^−2^
F17	3.01 × 10^−7^	4.60 × 10^−3^	3.02 × 10^−11^	7.30 × 10^−4^	3.34 × 10^−11^	3.02 × 10^−11^	6.01 × 10^−8^
F18	3.59 × 10^−5^	4.40 × 10^−3^	3.02 × 10^−11^	3.34 × 10^−11^	3.02 × 10^−11^	3.02 × 10^−11^	1.96 × 10^−10^
F19	6.20 × 10^−4^	3.63 × 10^−1^	3.02 × 10^−11^	1.33 × 10^−10^	3.02 × 10^−11^	3.02 × 10^−11^	9.23 × 10^−1^
F20	4.64 × 10^−5^	3.78 × 10^−2^	3.02 × 10^−11^	1.61 × 10^−6^	6.70 × 10^−11^	3.69 × 10^−11^	3.81 × 10^−7^
F21	1.49 × 10^−6^	1.90 × 10^−3^	3.02 × 10^−11^	9.51 × 10^−6^	3.02 × 10^−11^	3.02 × 10^−11^	1.55 × 10^−9^
F22	2.03 × 10^−9^	9.50 × 10^−3^	6.70 × 10^−11^	8.35 × 10^−8^	1.33 × 10^−10^	3.02 × 10^−11^	4.92 × 10^−1^
F23	3.18 × 10^−4^	1.91 × 10^−2^	3.02 × 10^−11^	5.86 × 10^−6^	3.02 × 10^−11^	3.02 × 10^−11^	8.89 × 10^−10^
F24	5.46 × 10^−9^	4.08 × 10^−5^	3.02 × 10^−11^	1.43 × 10^−8^	3.02 × 10^−11^	3.02 × 10^−11^	3.82 × 10^−10^
F25	8.77 × 10^−1^	2.68 × 10^−4^	3.02 × 10^−11^	3.02 × 10^−11^	3.02 × 10^−11^	3.02 × 10^−11^	5.30 × 10^−3^
F26	8.84 × 10^−7^	3.59 × 10^−5^	3.02 × 10^−11^	1.02 × 10^−5^	3.02 × 10^−11^	3.02 × 10^−11^	1.68 × 10^−4^
F27	4.20 × 10^−1^	9.35 × 10^−1^	3.02 × 10^−11^	6.36 × 10^−5^	5.49 × 10^−11^	3.02 × 10^−11^	9.03 × 10^−4^
F28	2.02 × 10^−8^	5.26 × 10^−4^	3.02 × 10^−11^	3.02 × 10^−11^	3.02 × 10^−11^	3.02 × 10^−11^	8.12 × 10^−4^
F29	2.19 × 10^−8^	4.71 × 10^−4^	3.02 × 10^−11^	1.31 × 10^−8^	3.02 × 10^−11^	3.02 × 10^−11^	2.20 × 10^−7^
F30	1.81 × 10^−1^	7.66 × 10^−5^	3.02 × 10^−11^	3.02 × 10^−11^	3.02 × 10^−11^	3.02 × 10^−11^	4.08 × 10^−5^

**Table A2:** Wilcoxon rank-sum test *p*-values for MALA versus each comparison algorithm on the CEC2014 30D benchmark functions.

Func.	ALA	EALA	SCA	GWO	WOA	HHO	MRFO
F1	3.37 × 10^−5^	5.97 × 10^−9^	3.02 × 10^−11^	4.08 × 10^−11^	3.02 × 10^−11^	3.02 × 10^−11^	1.25 × 10^−5^
F2	3.02 × 10^−11^	1.85 × 10^−8^	3.02 × 10^−11^	3.02 × 10^−11^	3.02 × 10^−11^	3.02 × 10^−11^	1.10 × 10^−8^
F3	7.74 × 10^−6^	8.00 × 10^−3^	3.02 × 10^−11^	3.02 × 10^−11^	3.02 × 10^−11^	3.02 × 10^−11^	2.64 × 10^−1^
F4	5.26 × 10^−4^	7.28 × 10^−1^	3.02 × 10^−11^	3.34 × 10^−11^	3.02 × 10^−11^	3.02 × 10^−11^	1.99 × 10^−2^
F5	5.01 × 10^−2^	4.12 × 10^−1^	8.15 × 10^−5^	1.25 × 10^−5^	5.80 × 10^−3^	2.39 × 10^−8^	1.49 × 10^−1^
F6	1.78 × 10^−10^	3.26 × 10^−7^	3.02 × 10^−11^	1.70 × 10^−8^	3.02 × 10^−11^	3.02 × 10^−11^	3.69 × 10^−11^
F7	3.02 × 10^−11^	3.02 × 10^−11^	3.02 × 10^−11^	3.02 × 10^−11^	3.02 × 10^−11^	3.02 × 10^−11^	3.02 × 10^−11^
F8	2.00 × 10^−5^	3.18 × 10^−1^	3.02 × 10^−11^	5.97 × 10^−5^	3.02 × 10^−11^	3.02 × 10^−11^	2.87 × 10^−10^
F9	4.04 × 10^−1^	3.18 × 10^−1^	3.02 × 10^−11^	8.56 × 10^−4^	3.02 × 10^−11^	3.02 × 10^−11^	3.82 × 10^−10^
F10	2.44 × 10^−9^	9.26 × 10^−9^	3.02 × 10^−11^	2.88 × 10^−6^	3.02 × 10^−11^	3.02 × 10^−11^	5.27 × 10^−5^
F11	2.68 × 10^−4^	2.96 × 10^−5^	3.02 × 10^−11^	6.15 × 10^−2^	6.12 × 10^−10^	3.69 × 10^−11^	7.28 × 10^−1^
F12	5.79 × 10^−1^	6.36 × 10^−5^	1.96 × 10^−10^	4.42 × 10^−6^	5.60 × 10^−3^	1.25 × 10^−5^	1.15 × 10^−1^
F13	5.30 × 10^−3^	5.46 × 10^−9^	3.02 × 10^−11^	4.62 × 10^−10^	9.92 × 10^−11^	3.02 × 10^−11^	3.82 × 10^−10^
F14	6.55 × 10^−4^	4.35 × 10^−5^	3.02 × 10^−11^	1.61 × 10^−10^	3.02 × 10^−11^	3.02 × 10^−11^	5.26 × 10^−4^
F15	1.49 × 10^−4^	2.80 × 10^−3^	3.02 × 10^−11^	1.21 × 10^−10^	3.02 × 10^−11^	3.02 × 10^−11^	2.25 × 10^−4^
F16	5.53 × 10^−8^	2.13 × 10^−5^	3.02 × 10^−11^	1.33 × 10^−2^	4.57 × 10^−9^	1.03 × 10^−6^	2.15 × 10^−6^
F17	4.94 × 10^−5^	2.15 × 10^−2^	3.02 × 10^−11^	5.49 × 10^−11^	3.02 × 10^−11^	3.02 × 10^−11^	1.11 × 10^−6^
F18	2.61 × 10^−10^	1.64 × 10^−5^	3.02 × 10^−11^	3.02 × 10^−11^	3.02 × 10^−11^	3.02 × 10^−11^	2.00 × 10^−6^
F19	4.11 × 10^−7^	2.68 × 10^−4^	3.02 × 10^−11^	3.02 × 10^−11^	3.02 × 10^−11^	3.02 × 10^−11^	1.07 × 10^−7^
F20	3.09 × 10^−6^	1.87 × 10^−5^	3.02 × 10^−11^	3.02 × 10^−11^	3.02 × 10^−11^	3.02 × 10^−11^	4.08 × 10^−11^
F21	3.83 × 10^−5^	1.15 × 10^−1^	3.02 × 10^−11^	3.02 × 10^−11^	3.02 × 10^−11^	3.02 × 10^−11^	6.12 × 10^−10^
F22	7.48 × 10^−2^	2.32 × 10^−2^	3.02 × 10^−11^	1.70 × 10^−2^	8.99 × 10^−11^	3.02 × 10^−11^	1.49 × 10^−6^
F23	1.12 × 10^−1^	3.96 × 10^−8^	3.02 × 10^−11^	3.02 × 10^−11^	3.02 × 10^−11^	1.21 × 10^−12^	1.21 × 10^−12^
F24	6.41 × 10^−1^	8.66 × 10^−5^	3.02 × 10^−11^	6.35 × 10^−2^	3.02 × 10^−11^	2.52 × 10^−11^	1.21 × 10^−12^
F25	5.49 × 10^−11^	1.25 × 10^−4^	3.02 × 10^−11^	8.48 × 10^−9^	6.63 × 10^−1^	1.21 × 10^−12^	1.21 × 10^−12^
F26	4.20 × 10^−3^	8.24 × 10^−2^	3.79 × 10^−1^	4.20 × 10^−3^	6.74 × 10^−1^	3.63 × 10^−1^	1.81 × 10^−1^
F27	1.06 × 10^−1^	5.94 × 10^−2^	7.39 × 10^−11^	6.12 × 10^−10^	9.92 × 10^−11^	1.21 × 10^−12^	1.21 × 10^−12^
F28	3.79 × 10^−1^	2.84 × 10^−4^	3.02 × 10^−11^	2.37 × 10^−10^	5.57 × 10^−10^	1.21 × 10^−12^	1.21 × 10^−12^
F29	2.03 × 10^−9^	2.13 × 10^−5^	3.02 × 10^−11^	5.09 × 10^−8^	7.38 × 10^−11^	4.56 × 10^−11^	1.72 × 10^−12^
F30	9.83 × 10^−8^	1.08 × 10^−2^	3.02 × 10^−11^	3.02 × 10^−11^	3.02 × 10^−11^	7.30 × 10^−3^	2.06 × 10^−1^

**Table A3:** Mean and Std of PSNR on multi-threshold segmentation (higher is better).

Image	TH	Type	MALA	ALA	EALA	SCA	GWO	WOA	HHO	MRFO
Airplane	2	Mean	**2.567 × 10^1^**	2.488 × 10^1^	2.516 × 10^1^	2.467 × 10^1^	2.566 × 10^1^	2.514 × 10^1^	2.451 × 10^1^	2.533 × 10^1^
		Std	7.149 × 10^−5^	5.619 × 10^−1^	5.983 × 10^−1^	5.699 × 10^−1^	3.262 × 10^−2^	5.057 × 10^−1^	5.725 × 10^−1^	5.546 × 10^−1^
	3	Mean	**2.781 × 10^1^**	2.697 × 10^1^	2.679 × 10^1^	2.646 × 10^1^	2.758 × 10^1^	2.672 × 10^1^	2.662 × 10^1^	2.674 × 10^1^
		Std	6.782 × 10^−3^	3.548 × 10^−1^	3.699 × 10^−1^	1.879 × 10^−1^	2.509 × 10^−1^	3.532 × 10^−1^	3.137 × 10^−1^	4.937 × 10^−1^
	4	Mean	**2.961 × 10^1^**	2.838 × 10^1^	2.833 × 10^1^	2.769 × 10^1^	2.863 × 10^1^	2.780 × 10^1^	2.779 × 10^1^	2.834 × 10^1^
		Std	2.427 × 10^−2^	4.489 × 10^−1^	5.955 × 10^−1^	2.789 × 10^−1^	5.108 × 10^−1^	5.706 × 10^−1^	5.226 × 10^−1^	7.843 × 10^−1^
	5	Mean	**3.115 × 10^1^**	2.905 × 10^1^	2.935 × 10^1^	2.864 × 10^1^	2.974 × 10^1^	2.868 × 10^1^	2.880 × 10^1^	2.959 × 10^1^
		Std	5.921 × 10^−2^	4.578 × 10^−1^	4.075 × 10^−1^	1.617 × 10^−1^	4.574 × 10^−1^	3.002 × 10^−1^	4.158 × 10^−1^	8.524 × 10^−1^
Baboon	2	Mean	**2.283 × 10^1^**	2.209 × 10^1^	2.238 × 10^1^	2.174 × 10^1^	2.283 × 10^1^	2.250 × 10^1^	2.204 × 10^1^	2.275 × 10^1^
		Std	1.485 × 10^−4^	6.212 × 10^−1^	5.401 × 10^−1^	5.411 × 10^−1^	5.121 × 10^−4^	4.957 × 10^−1^	4.971 × 10^−1^	2.339 × 10^−1^
	3	Mean	**2.531 × 10^1^**	2.395 × 10^1^	2.390 × 10^1^	2.380 × 10^1^	2.502 × 10^1^	2.391 × 10^1^	2.383 × 10^1^	2.401 × 10^1^
		Std	4.458 × 10^−3^	5.411 × 10^−1^	4.159 × 10^−1^	2.588 × 10^−1^	4.411 × 10^−1^	5.049 × 10^−1^	5.025 × 10^−1^	6.740 × 10^−1^
	4	Mean	**2.721 × 10^1^**	2.563 × 10^1^	2.555 × 10^1^	2.559 × 10^1^	2.652 × 10^1^	2.546 × 10^1^	2.552 × 10^1^	2.587 × 10^1^
		Std	1.231 × 10^−2^	3.537 × 10^−1^	2.653 × 10^−1^	1.845 × 10^−1^	4.917 × 10^−1^	0.000 × 10^0^	1.899 × 10^−1^	5.045 × 10^−1^
	5	Mean	**2.872 × 10^1^**	2.706 × 10^1^	2.703 × 10^1^	2.700 × 10^1^	2.755 × 10^1^	2.699 × 10^1^	2.703 × 10^1^	2.707 × 10^1^
		Std	3.322 × 10^−2^	2.449 × 10^−1^	2.704 × 10^−1^	9.693 × 10^−2^	2.125 × 10^−1^	9.646 × 10^−2^	2.105 × 10^−1^	2.473 × 10^−1^
House	2	Mean	**2.588 × 10^1^**	2.575 × 10^1^	2.578 × 10^1^	2.496 × 10^1^	2.583 × 10^1^	2.558 × 10^1^	2.475 × 10^1^	2.581 × 10^1^
		Std	3.271 × 10^−2^	1.973 × 10^−1^	2.626 × 10^−1^	4.295 × 10^−1^	1.697 × 10^−1^	3.221 × 10^−1^	6.523 × 10^−1^	1.454 × 10^−1^
	3	Mean	**2.836 × 10^1^**	2.684 × 10^1^	2.682 × 10^1^	2.659 × 10^1^	2.786 × 10^1^	2.683 × 10^1^	2.658 × 10^1^	2.702 × 10^1^
		Std	6.485 × 10^−4^	2.988 × 10^−1^	3.763 × 10^−1^	2.509 × 10^−1^	4.547 × 10^−1^	4.615 × 10^−1^	4.664 × 10^−1^	6.864 × 10^−1^
	4	Mean	**2.976 × 10^1^**	2.817 × 10^1^	2.879 × 10^1^	2.743 × 10^1^	2.932 × 10^1^	2.779 × 10^1^	2.760 × 10^1^	2.885 × 10^1^
		Std	6.282 × 10^−2^	8.088 × 10^−1^	9.698 × 10^−1^	2.303 × 10^−1^	6.292 × 10^−1^	9.124 × 10^−1^	6.160 × 10^−1^	1.101 × 10^0^
	5	Mean	**3.138 × 10^1^**	2.971 × 10^1^	2.973 × 10^1^	2.967 × 10^1^	3.029 × 10^1^	2.968 × 10^1^	2.967 × 10^1^	2.990 × 10^1^
		Std	6.877 × 10^−2^	2.333 × 10^−1^	2.398 × 10^−1^	5.072 × 10^−2^	6.109 × 10^−1^	9.857 × 10^−2^	8.422 × 10^−2^	4.396 × 10^−1^
Lake	2	Mean	**2.356 × 10^1^**	2.313 × 10^1^	2.326 × 10^1^	2.270 × 10^1^	2.355 × 10^1^	2.323 × 10^1^	2.271 × 10^1^	2.347 × 10^1^
		Std	1.076 × 10^−5^	5.480 × 10^−1^	4.841 × 10^−1^	3.510 × 10^−1^	2.962 × 10^−2^	4.849 × 10^−1^	5.239 × 10^−1^	2.820 × 10^−1^
	3	Mean	**2.576 × 10^1^**	2.490 × 10^1^	2.483 × 10^1^	2.475 × 10^1^	2.568 × 10^1^	2.479 × 10^1^	2.457 × 10^1^	2.472 × 10^1^
		Std	1.800 × 10^−3^	3.521 × 10^−1^	3.055 × 10^−1^	1.033 × 10^−1^	1.689 × 10^−1^	2.464 × 10^−1^	3.859 × 10^−1^	3.204 × 10^−2^
	4	Mean	**2.759 × 10^1^**	2.679 × 10^1^	2.652 × 10^1^	2.646 × 10^1^	2.732 × 10^1^	2.644 × 10^1^	2.631 × 10^1^	2.674 × 10^1^
		Std	8.721 × 10^−2^	5.664 × 10^−1^	4.465 × 10^−1^	2.691 × 10^−1^	2.802 × 10^−1^	2.693 × 10^−1^	3.244 × 10^−2^	5.525 × 10^−1^
	5	Mean	**2.935 × 10^1^**	2.773 × 10^1^	2.778 × 10^1^	2.767 × 10^1^	2.854 × 10^1^	2.783 × 10^1^	2.766 × 10^1^	2.778 × 10^1^
		Std	4.887 × 10^−2^	2.505 × 10^−1^	3.232 × 10^−1^	3.020 × 10^−2^	3.594 × 10^−1^	3.686 × 10^−1^	1.094 × 10^−14^	2.623 × 10^−1^
Car	2	Mean	**2.300 × 10^1^**	2.249 × 10^1^	2.254 × 10^1^	2.207 × 10^1^	2.298 × 10^1^	2.253 × 10^1^	2.209 × 10^1^	2.252 × 10^1^
		Std	1.094 × 10^−14^	1.371 × 10^−1^	2.060 × 10^−1^	5.265 × 10^−1^	6.342 × 10^−2^	1.892 × 10^−1^	5.325 × 10^−1^	2.004 × 10^−1^
	3	Mean	**2.554 × 10^1^**	2.446 × 10^1^	2.448 × 10^1^	2.431 × 10^1^	2.531 × 10^1^	2.444 × 10^1^	2.426 × 10^1^	2.450 × 10^1^
		Std	3.038 × 10^−3^	2.671 × 10^−1^	2.825 × 10^−1^	2.020 × 10^−1^	2.577 × 10^−1^	2.187 × 10^−1^	4.312 × 10^−1^	3.555 × 10^−1^
	4	Mean	**2.739 × 10^1^**	2.616 × 10^1^	2.618 × 10^1^	2.617 × 10^1^	2.672 × 10^1^	2.616 × 10^1^	2.616 × 10^1^	2.616 × 10^1^
		Std	2.481 × 10^−2^	1.094 × 10^−14^	7.416 × 10^−2^	3.314 × 10^−2^	3.887 × 10^−1^	1.094 × 10^−14^	1.094 × 10^−14^	1.094 × 10^−14^
	5	Mean	**2.910 × 10^1^**	2.704 × 10^1^	2.721 × 10^1^	2.698 × 10^1^	2.777 × 10^1^	2.703 × 10^1^	2.695 × 10^1^	2.729 × 10^1^
		Std	9.747 × 10^−2^	2.551 × 10^−1^	5.107 × 10^−1^	1.016 × 10^−1^	4.714 × 10^−1^	3.147 × 10^−1^	1.116 × 10^−1^	4.545 × 10^−1^
Beans	2	Mean	**2.712 × 10^1^**	2.641 × 10^1^	2.658 × 10^1^	2.609 × 10^1^	2.711 × 10^1^	2.642 × 10^1^	2.598 × 10^1^	2.709 × 10^1^
		Std	3.645 × 10^−15^	8.179 × 10^−1^	8.188 × 10^−1^	5.638 × 10^−1^	2.264 × 10^−2^	7.446 × 10^−1^	5.260 × 10^−1^	1.151 × 10^−1^
	3	Mean	**2.919 × 10^1^**	2.830 × 10^1^	2.882 × 10^1^	2.722 × 10^1^	2.902 × 10^1^	2.829 × 10^1^	2.736 × 10^1^	2.907 × 10^1^
		Std	3.767 × 10^−2^	9.135 × 10^−1^	5.092 × 10^−1^	5.075 × 10^−1^	3.150 × 10^−1^	8.987 × 10^−1^	6.346 × 10^−1^	2.968 × 10^−1^
	4	Mean	**3.096 × 10^1^**	2.901 × 10^1^	2.971 × 10^1^	2.833 × 10^1^	3.038 × 10^1^	2.846 × 10^1^	2.861 × 10^1^	3.023 × 10^1^
		Std	4.165 × 10^−2^	8.302 × 10^−1^	1.189 × 10^0^	2.631 × 10^−1^	6.527 × 10^−1^	7.507 × 10^−1^	5.440 × 10^−1^	1.006 × 10^0^
	5	Mean	**3.246 × 10^1^**	3.022 × 10^1^	3.040 × 10^1^	2.910 × 10^1^	3.118 × 10^1^	3.019 × 10^1^	2.932 × 10^1^	3.168 × 10^1^
		Std	7.079 × 10^−2^	9.333 × 10^−1^	1.382 × 10^0^	3.003 × 10^−1^	9.129 × 10^−1^	9.526 × 10^−1^	6.740 × 10^−1^	9.729 × 10^−1^

**Table A4:** Mean and Std of SSIM on multi-threshold segmentation (higher is better).

Image	TH	Type	MALA	ALA	EALA	SCA	GWO	WOA	HHO	MRFO
Airplane	2	Mean	**8.708 × 10^−1^**	8.494 × 10^−1^	8.568 × 10^−1^	8.262 × 10^−1^	8.692 × 10^−1^	8.523 × 10^−1^	8.279 × 10^−1^	8.612 × 10^−1^
		Std	1.670 × 10^−4^	1.559 × 10^−2^	1.646 × 10^−2^	3.313 × 10^−2^	3.282 × 10^−3^	1.485 × 10^−2^	2.798 × 10^−2^	1.514 × 10^−2^
	3	Mean	**8.671 × 10^−1^**	8.554 × 10^−1^	8.519 × 10^−1^	8.463 × 10^−1^	8.651 × 10^−1^	8.514 × 10^−1^	8.478 × 10^−1^	8.510 × 10^−1^
		Std	3.004 × 10^−3^	6.464 × 10^−3^	5.934 × 10^−3^	7.763 × 10^−3^	8.722 × 10^−3^	1.046 × 10^−2^	7.070 × 10^−3^	8.797 × 10^−3^
	4	Mean	**8.915 × 10^−1^**	8.567 × 10^−1^	8.520 × 10^−1^	8.323 × 10^−1^	8.658 × 10^−1^	8.379 × 10^−1^	8.395 × 10^−1^	8.546 × 10^−1^
		Std	8.190 × 10^−3^	1.530 × 10^−2^	1.695 × 10^−2^	7.714 × 10^−3^	2.163 × 10^−2^	2.094 × 10^−2^	2.108 × 10^−2^	2.656 × 10^−2^
	5	Mean	**9.161 × 10^−1^**	8.629 × 10^−1^	8.636 × 10^−1^	8.608 × 10^−1^	8.632 × 10^−1^	8.638 × 10^−1^	8.712 × 10^−1^	8.707 × 10^−1^
		Std	4.574 × 10^−3^	1.164 × 10^−2^	9.567 × 10^−3^	1.350 × 10^−2^	1.567 × 10^−2^	1.400 × 10^−2^	1.028 × 10^−2^	2.572 × 10^−2^
Baboon	2	Mean	8.023 × 10^−1^	7.730 × 10^−1^	7.840 × 10^−1^	7.654 × 10^−1^	**8.028 × 10^−1^**	7.917 × 10^−1^	7.809 × 10^−1^	7.993 × 10^−1^
		Std	1.309 × 10^−4^	2.358 × 10^−2^	2.124 × 10^−2^	1.948 × 10^−2^	6.827 × 10^−4^	1.946 × 10^−2^	1.935 × 10^−2^	9.413 × 10^−3^
	3	Mean	**8.710 × 10^−1^**	8.282 × 10^−1^	8.257 × 10^−1^	8.245 × 10^−1^	8.623 × 10^−1^	8.266 × 10^−1^	8.301 × 10^−1^	8.304 × 10^−1^
		Std	3.352 × 10^−4^	1.620 × 10^−2^	1.042 × 10^−2^	7.816 × 10^−3^	1.244 × 10^−2^	1.522 × 10^−2^	1.671 × 10^−2^	2.090 × 10^−2^
	4	Mean	**9.085 × 10^−1^**	8.737 × 10^−1^	8.724 × 10^−1^	8.730 × 10^−1^	8.928 × 10^−1^	8.711 × 10^−1^	8.723 × 10^−1^	8.782 × 10^−1^
		Std	5.695 × 10^−4^	5.471 × 10^−3^	4.098 × 10^−3^	2.873 × 10^−3^	1.063 × 10^−2^	3.417 × 10^−16^	3.885 × 10^−3^	9.715 × 10^−3^
	5	Mean	**9.327 × 10^−1^**	9.011 × 10^−1^	9.005 × 10^−1^	9.002 × 10^−1^	9.095 × 10^−1^	8.999 × 10^−1^	9.010 × 10^−1^	9.011 × 10^−1^
		Std	8.877 × 10^−4^	3.848 × 10^−3^	4.186 × 10^−3^	1.581 × 10^−3^	3.031 × 10^−3^	1.407 × 10^−3^	4.641 × 10^−3^	3.412 × 10^−3^
House	2	Mean	**8.978 × 10^−1^**	8.951 × 10^−1^	8.958 × 10^−1^	8.854 × 10^−1^	8.972 × 10^−1^	8.931 × 10^−1^	8.775 × 10^−1^	8.978 × 10^−1^
		Std	2.363 × 10^−3^	3.956 × 10^−3^	3.449 × 10^−3^	1.219 × 10^−2^	3.260 × 10^−3^	7.089 × 10^−3^	1.897 × 10^−2^	5.380 × 10^−3^
	3	Mean	**9.360 × 10^−1^**	9.052 × 10^−1^	9.053 × 10^−1^	9.037 × 10^−1^	9.265 × 10^−1^	9.050 × 10^−1^	9.066 × 10^−1^	9.086 × 10^−1^
		Std	1.200 × 10^−4^	6.397 × 10^−3^	9.647 × 10^−3^	5.419 × 10^−3^	9.126 × 10^−3^	9.738 × 10^−3^	8.163 × 10^−3^	1.397 × 10^−2^
	4	Mean	**9.527 × 10^−1^**	9.309 × 10^−1^	9.377 × 10^−1^	9.240 × 10^−1^	9.454 × 10^−1^	9.259 × 10^−1^	9.243 × 10^−1^	9.382 × 10^−1^
		Std	1.891 × 10^−3^	1.173 × 10^−2^	1.275 × 10^−2^	4.771 × 10^−3^	8.281 × 10^−3^	1.361 × 10^−2^	8.765 × 10^−3^	1.515 × 10^−2^
	5	Mean	**9.643 × 10^−1^**	9.422 × 10^−1^	9.425 × 10^−1^	9.419 × 10^−1^	9.507 × 10^−1^	9.417 × 10^−1^	9.419 × 10^−1^	9.445 × 10^−1^
		Std	1.123 × 10^−3^	2.919 × 10^−3^	3.022 × 10^−3^	1.345 × 10^−3^	6.680 × 10^−3^	5.715 × 10^−4^	1.497 × 10^−3^	5.398 × 10^−3^
Lake	2	Mean	**8.611 × 10^−1^**	8.511 × 10^−1^	8.549 × 10^−1^	8.396 × 10^−1^	8.596 × 10^−1^	8.500 × 10^−1^	8.423 × 10^−1^	8.589 × 10^−1^
		Std	9.088 × 10^−5^	1.413 × 10^−2^	1.119 × 10^−2^	1.193 × 10^−2^	2.109 × 10^−3^	1.379 × 10^−2^	1.443 × 10^−2^	8.108 × 10^−3^
	3	Mean	**8.919 × 10^−1^**	8.760 × 10^−1^	8.748 × 10^−1^	8.739 × 10^−1^	8.897 × 10^−1^	8.739 × 10^−1^	8.701 × 10^−1^	8.726 × 10^−1^
		Std	9.395 × 10^−4^	7.886 × 10^−3^	8.035 × 10^−3^	3.753 × 10^−3^	6.580 × 10^−3^	5.744 × 10^−3^	9.272 × 10^−3^	3.143 × 10^−3^
	4	Mean	**9.298 × 10^−1^**	9.172 × 10^−1^	9.123 × 10^−1^	9.113 × 10^−1^	9.267 × 10^−1^	9.107 × 10^−1^	9.086 × 10^−1^	9.164 × 10^−1^
		Std	6.079 × 10^−3^	1.026 × 10^−2^	8.043 × 10^−3^	5.597 × 10^−3^	5.155 × 10^−3^	4.545 × 10^−3^	2.426 × 10^−4^	9.796 × 10^−3^
	5	Mean	**9.490 × 10^−1^**	9.223 × 10^−1^	9.231 × 10^−1^	9.213 × 10^−1^	9.359 × 10^−1^	9.239 × 10^−1^	9.209 × 10^−1^	9.231 × 10^−1^
		Std	8.724 × 10^−4^	4.332 × 10^−3^	5.432 × 10^−3^	1.143 × 10^−3^	6.028 × 10^−3^	6.131 × 10^−3^	2.278 × 10^−16^	4.749 × 10^−3^
Car	2	Mean	**7.261 × 10^−1^**	6.985 × 10^−1^	7.010 × 10^−1^	7.019 × 10^−1^	7.254 × 10^−1^	7.042 × 10^−1^	7.050 × 10^−1^	7.004 × 10^−1^
		Std	2.278 × 10^−16^	7.656 × 10^−3^	1.126 × 10^−2^	3.672 × 10^−2^	2.194 × 10^−3^	2.237 × 10^−2^	4.104 × 10^−2^	1.265 × 10^−2^
	3	Mean	**8.623 × 10^−1^**	8.194 × 10^−1^	8.196 × 10^−1^	8.181 × 10^−1^	8.547 × 10^−1^	8.177 × 10^−1^	8.113 × 10^−1^	8.205 × 10^−1^
		Std	2.817 × 10^−3^	1.193 × 10^−2^	1.063 × 10^−2^	1.320 × 10^−2^	9.880 × 10^−3^	4.786 × 10^−3^	2.503 × 10^−2^	1.334 × 10^−2^
	4	Mean	**8.957 × 10^−1^**	8.734 × 10^−1^	8.736 × 10^−1^	8.732 × 10^−1^	8.851 × 10^−1^	8.734 × 10^−1^	8.734 × 10^−1^	8.734 × 10^−1^
		Std	1.974 × 10^−3^	1.139 × 10^−16^	4.856 × 10^−4^	1.637 × 10^−3^	1.013 × 10^−2^	1.139 × 10^−16^	1.139 × 10^−16^	1.139 × 10^−16^
	5	Mean	**9.233 × 10^−1^**	8.412 × 10^−1^	8.550 × 10^−1^	8.376 × 10^−1^	8.895 × 10^−1^	8.368 × 10^−1^	8.318 × 10^−1^	8.730 × 10^−1^
		Std	1.289 × 10^−3^	3.039 × 10^−2^	4.088 × 10^−2^	2.319 × 10^−2^	2.837 × 10^−2^	2.679 × 10^−2^	1.669 × 10^−2^	4.093 × 10^−2^
Beans	2	Mean	**9.489 × 10^−1^**	9.339 × 10^−1^	9.380 × 10^−1^	9.389 × 10^−1^	9.485 × 10^−1^	9.375 × 10^−1^	9.416 × 10^−1^	9.485 × 10^−1^
		Std	1.139 × 10^−16^	1.797 × 10^−2^	1.803 × 10^−2^	9.511 × 10^−3^	6.306 × 10^−4^	1.776 × 10^−2^	7.372 × 10^−3^	1.918 × 10^−3^
	3	Mean	**9.632 × 10^−1^**	9.518 × 10^−1^	9.599 × 10^−1^	9.425 × 10^−1^	9.620 × 10^−1^	9.522 × 10^−1^	9.511 × 10^−1^	9.621 × 10^−1^
		Std	8.500 × 10^−4^	1.703 × 10^−2^	6.617 × 10^−3^	1.416 × 10^−2^	2.430 × 10^−3^	1.861 × 10^−2^	1.152 × 10^−2^	3.277 × 10^−3^
	4	Mean	**9.739 × 10^−1^**	9.614 × 10^−1^	9.654 × 10^−1^	9.566 × 10^−1^	9.706 × 10^−1^	9.592 × 10^−1^	9.598 × 10^−1^	9.689 × 10^−1^
		Std	6.383 × 10^−4^	5.447 × 10^−3^	8.169 × 10^−3^	4.617 × 10^−3^	3.551 × 10^−3^	4.687 × 10^−3^	4.548 × 10^−3^	6.409 × 10^−3^
	5	Mean	**9.803 × 10^−1^**	9.696 × 10^−1^	9.692 × 10^−1^	9.632 × 10^−1^	9.744 × 10^−1^	9.692 × 10^−1^	9.647 × 10^−1^	9.769 × 10^−1^
		Std	1.171 × 10^−3^	7.200 × 10^−3^	8.252 × 10^−3^	4.101 × 10^−3^	4.799 × 10^−3^	4.834 × 10^−3^	3.747 × 10^−3^	4.585 × 10^−3^

**Table A5:** Mean and Std of FSIM on multi-threshold segmentation (higher is better).

Image	TH	Type	MALA	ALA	EALA	SCA	GWO	WOA	HHO	MRFO
Airplane	2	Mean	**8.799 × 10^−1^**	8.668 × 10^−1^	8.716 × 10^−1^	8.725 × 10^−1^	8.798 × 10^−1^	8.723 × 10^−1^	8.679 × 10^−1^	8.749 × 10^−1^
		Std	2.510 × 10^−4^	1.075 × 10^−2^	1.091 × 10^−2^	1.408 × 10^−2^	1.344 × 10^−3^	1.190 × 10^−2^	1.598 × 10^−2^	9.184 × 10^−3^
	3	Mean	**9.236 × 10^−1^**	8.913 × 10^−1^	8.865 × 10^−1^	8.857 × 10^−1^	9.139 × 10^−1^	8.863 × 10^−1^	8.895 × 10^−1^	8.903 × 10^−1^
		Std	1.956 × 10^−3^	1.291 × 10^−2^	9.090 × 10^−3^	4.381 × 10^−3^	1.062 × 10^−2^	6.634 × 10^−3^	1.094 × 10^−2^	1.472 × 10^−2^
	4	Mean	**9.296 × 10^−1^**	9.067 × 10^−1^	9.105 × 10^−1^	9.064 × 10^−1^	9.162 × 10^−1^	9.082 × 10^−1^	9.131 × 10^−1^	9.106 × 10^−1^
		Std	8.628 × 10^−3^	1.544 × 10^−3^	9.145 × 10^−3^	2.121 × 10^−3^	9.232 × 10^−3^	6.618 × 10^−3^	1.093 × 10^−2^	7.836 × 10^−3^
	5	Mean	**9.376 × 10^−1^**	9.185 × 10^−1^	9.213 × 10^−1^	9.170 × 10^−1^	9.325 × 10^−1^	9.167 × 10^−1^	9.226 × 10^−1^	9.276 × 10^−1^
		Std	6.967 × 10^−3^	3.364 × 10^−3^	5.360 × 10^−3^	4.759 × 10^−3^	6.184 × 10^−3^	3.505 × 10^−3^	1.065 × 10^−2^	1.070 × 10^−2^
Baboon	2	Mean	8.518 × 10^−1^	8.183 × 10^−1^	8.355 × 10^−1^	8.086 × 10^−1^	**8.523 × 10^−1^**	8.370 × 10^−1^	8.288 × 10^−1^	8.493 × 10^−1^
		Std	1.323 × 10^−4^	3.087 × 10^−2^	2.576 × 10^−2^	3.366 × 10^−2^	1.017 × 10^−3^	2.668 × 10^−2^	2.102 × 10^−2^	7.767 × 10^−3^
	3	Mean	**9.011 × 10^−1^**	8.598 × 10^−1^	8.610 × 10^−1^	8.586 × 10^−1^	8.955 × 10^−1^	8.602 × 10^−1^	8.590 × 10^−1^	8.599 × 10^−1^
		Std	7.784 × 10^−4^	2.586 × 10^−2^	2.438 × 10^−2^	1.852 × 10^−2^	1.347 × 10^−2^	2.269 × 10^−2^	2.548 × 10^−2^	2.433 × 10^−2^
	4	Mean	**9.311 × 10^−1^**	9.060 × 10^−1^	9.029 × 10^−1^	9.041 × 10^−1^	9.244 × 10^−1^	8.998 × 10^−1^	9.019 × 10^−1^	9.128 × 10^−1^
		Std	1.004 × 10^−3^	1.283 × 10^−2^	9.545 × 10^−3^	6.868 × 10^−3^	9.594 × 10^−3^	4.556 × 10^−16^	6.846 × 10^−3^	1.418 × 10^−2^
	5	Mean	**9.502 × 10^−1^**	9.267 × 10^−1^	9.254 × 10^−1^	9.255 × 10^−1^	9.401 × 10^−1^	9.246 × 10^−1^	9.258 × 10^−1^	9.275 × 10^−1^
		Std	1.614 × 10^−3^	7.610 × 10^−3^	6.600 × 10^−3^	3.591 × 10^−3^	9.391 × 10^−3^	3.046 × 10^−3^	5.795 × 10^−3^	7.876 × 10^−3^
House	2	Mean	**8.704 × 10^−1^**	8.702 × 10^−1^	8.675 × 10^−1^	8.615 × 10^−1^	8.660 × 10^−1^	8.644 × 10^−1^	8.625 × 10^−1^	8.690 × 10^−1^
		Std	8.515 × 10^−3^	8.488 × 10^−3^	9.010 × 10^−3^	1.457 × 10^−2^	7.402 × 10^−3^	8.093 × 10^−3^	1.621 × 10^−2^	7.958 × 10^−3^
	3	Mean	**8.957 × 10^−1^**	8.796 × 10^−1^	8.798 × 10^−1^	8.800 × 10^−1^	8.907 × 10^−1^	8.799 × 10^−1^	8.821 × 10^−1^	8.806 × 10^−1^
		Std	3.137 × 10^−4^	5.653 × 10^−3^	8.401 × 10^−3^	1.273 × 10^−2^	7.409 × 10^−3^	8.973 × 10^−3^	1.052 × 10^−2^	7.222 × 10^−3^
	4	Mean	**9.203 × 10^−1^**	9.167 × 10^−1^	9.180 × 10^−1^	9.088 × 10^−1^	9.203 × 10^−1^	9.138 × 10^−1^	9.110 × 10^−1^	9.159 × 10^−1^
		Std	1.203 × 10^−2^	8.792 × 10^−3^	8.839 × 10^−3^	1.155 × 10^−2^	1.079 × 10^−2^	8.690 × 10^−3^	1.115 × 10^−2^	8.695 × 10^−3^
	5	Mean	**9.472 × 10^−1^**	9.325 × 10^−1^	9.330 × 10^−1^	9.324 × 10^−1^	9.341 × 10^−1^	9.324 × 10^−1^	9.323 × 10^−1^	9.323 × 10^−1^
		Std	7.800 × 10^−3^	3.551 × 10^−4^	1.468 × 10^−3^	1.923 × 10^−3^	6.092 × 10^−3^	7.608 × 10^−4^	1.534 × 10^−3^	1.780 × 10^−3^
Lake	2	Mean	8.398 × 10^−1^	8.399 × 10^−1^	8.387 × 10^−1^	8.371 × 10^−1^	8.398 × 10^−1^	**8.437 × 10^−1^**	8.359 × 10^−1^	8.404 × 10^−1^
		Std	7.974 × 10^−5^	8.610 × 10^−3^	7.304 × 10^−3^	1.419 × 10^−2^	1.075 × 10^−3^	5.923 × 10^−3^	1.354 × 10^−2^	3.134 × 10^−3^
	3	Mean	**8.955 × 10^−1^**	8.811 × 10^−1^	8.808 × 10^−1^	8.773 × 10^−1^	8.936 × 10^−1^	8.801 × 10^−1^	8.774 × 10^−1^	8.791 × 10^−1^
		Std	4.936 × 10^−4^	1.272 × 10^−3^	9.589 × 10^−4^	6.128 × 10^−3^	4.615 × 10^−3^	3.182 × 10^−3^	1.105 × 10^−2^	4.106 × 10^−3^
	4	Mean	**9.116 × 10^−1^**	9.062 × 10^−1^	9.045 × 10^−1^	9.031 × 10^−1^	9.089 × 10^−1^	9.030 × 10^−1^	9.033 × 10^−1^	9.052 × 10^−1^
		Std	2.744 × 10^−3^	4.327 × 10^−3^	3.052 × 10^−3^	2.872 × 10^−3^	4.581 × 10^−3^	8.862 × 10^−4^	4.049 × 10^−4^	3.739 × 10^−3^
	5	Mean	**9.337 × 10^−1^**	9.276 × 10^−1^	9.279 × 10^−1^	9.272 × 10^−1^	9.311 × 10^−1^	9.282 × 10^−1^	9.273 × 10^−1^	9.276 × 10^−1^
		Std	1.847 × 10^−3^	1.641 × 10^−3^	2.084 × 10^−3^	2.300 × 10^−4^	3.453 × 10^−3^	2.379 × 10^−3^	1.139 × 10^−16^	1.485 × 10^−3^
Car	2	Mean	**8.547 × 10^−1^**	8.454 × 10^−1^	8.465 × 10^−1^	8.400 × 10^−1^	8.540 × 10^−1^	8.464 × 10^−1^	8.464 × 10^−1^	8.453 × 10^−1^
		Std	0.000 × 10^0^	4.137 × 10^−3^	4.719 × 10^−3^	1.582 × 10^−2^	1.087 × 10^−3^	5.520 × 10^−3^	1.062 × 10^−2^	3.654 × 10^−3^
	3	Mean	**8.945 × 10^−1^**	8.836 × 10^−1^	8.838 × 10^−1^	8.818 × 10^−1^	8.902 × 10^−1^	8.835 × 10^−1^	8.811 × 10^−1^	8.845 × 10^−1^
		Std	1.599 × 10^−3^	3.054 × 10^−3^	2.755 × 10^−3^	6.341 × 10^−3^	4.844 × 10^−3^	2.708 × 10^−3^	7.565 × 10^−3^	4.548 × 10^−3^
	4	Mean	**9.220 × 10^−1^**	9.173 × 10^−1^	9.174 × 10^−1^	9.172 × 10^−1^	9.195 × 10^−1^	9.173 × 10^−1^	9.173 × 10^−1^	9.173 × 10^−1^
		Std	1.547 × 10^−3^	2.278 × 10^−16^	2.831 × 10^−4^	2.508 × 10^−3^	4.634 × 10^−3^	2.278 × 10^−16^	2.278 × 10^−16^	2.278 × 10^−16^
	5	Mean	**9.418 × 10^−1^**	9.306 × 10^−1^	9.314 × 10^−1^	9.299 × 10^−1^	9.336 × 10^−1^	9.312 × 10^−1^	9.303 × 10^−1^	9.305 × 10^−1^
		Std	1.260 × 10^−3^	1.905 × 10^−3^	3.385 × 10^−3^	2.722 × 10^−3^	5.250 × 10^−3^	1.446 × 10^−3^	2.161 × 10^−3^	3.612 × 10^−3^
Beans	2	Mean	9.220 × 10^−1^	**9.239 × 10^−1^**	9.229 × 10^−1^	9.177 × 10^−1^	9.218 × 10^−1^	9.223 × 10^−1^	9.160 × 10^−1^	9.224 × 10^−1^
		Std	2.278 × 10^−16^	3.864 × 10^−3^	3.974 × 10^−3^	7.548 × 10^−3^	3.571 × 10^−4^	4.623 × 10^−3^	7.350 × 10^−3^	1.952 × 10^−3^
	3	Mean	**9.380 × 10^−1^**	9.375 × 10^−1^	9.379 × 10^−1^	9.351 × 10^−1^	9.359 × 10^−1^	9.373 × 10^−1^	9.315 × 10^−1^	9.372 × 10^−1^
		Std	1.168 × 10^−3^	2.289 × 10^−3^	2.250 × 10^−3^	6.158 × 10^−3^	3.774 × 10^−3^	4.291 × 10^−3^	6.309 × 10^−3^	1.675 × 10^−3^
	4	Mean	**9.516 × 10^−1^**	9.458 × 10^−1^	9.482 × 10^−1^	9.418 × 10^−1^	9.510 × 10^−1^	9.423 × 10^−1^	9.440 × 10^−1^	9.488 × 10^−1^
		Std	1.035 × 10^−3^	4.348 × 10^−3^	5.055 × 10^−3^	3.417 × 10^−3^	2.513 × 10^−3^	2.296 × 10^−3^	5.517 × 10^−3^	3.687 × 10^−3^
	5	Mean	**9.624 × 10^−1^**	9.554 × 10^−1^	9.566 × 10^−1^	9.535 × 10^−1^	9.583 × 10^−1^	9.543 × 10^−1^	9.509 × 10^−1^	9.597 × 10^−1^
		Std	1.484 × 10^−3^	3.251 × 10^−3^	3.398 × 10^−3^	1.990 × 10^−3^	3.826 × 10^−3^	2.794 × 10^−3^	6.188 × 10^−3^	4.081 × 10^−3^

**Table A6:** Mean, Std, and AvgRank of PSNR for remote sensing images under multi-threshold segmentation.

Image	TH	Type	MALA	ALA	EALA	SCA	GWO	WOA	HHO	MRFO
Beach	2	Mean	**2.415 × 10^1^**	2.403 × 10^1^	2.414 × 10^1^	2.307 × 10^1^	2.415 × 10^1^	2.363 × 10^1^	2.302 × 10^1^	2.412 × 10^1^
		Std	7.290 × 10^−15^	4.269 × 10^−1^	4.277 × 10^−2^	3.620 × 10^−1^	1.101 × 10^−3^	5.616 × 10^−1^	5.837 × 10^−1^	1.721 × 10^−1^
	3	Mean	**2.675 × 10^1^**	2.570 × 10^1^	2.643 × 10^1^	2.417 × 10^1^	2.659 × 10^1^	2.560 × 10^1^	2.458 × 10^1^	2.648 × 10^1^
		Std	3.707 × 10^−2^	1.049 × 10^0^	6.207 × 10^−1^	4.111 × 10^−1^	3.421 × 10^−1^	7.321 × 10^−1^	7.734 × 10^−1^	5.230 × 10^−1^
	4	Mean	**2.914 × 10^1^**	2.651 × 10^1^	2.700 × 10^1^	2.589 × 10^1^	2.776 × 10^1^	2.681 × 10^1^	2.598 × 10^1^	2.749 × 10^1^
		Std	1.124 × 10^−2^	5.094 × 10^−1^	1.048 × 10^0^	1.779 × 10^−1^	1.009 × 10^0^	1.080 × 10^0^	5.112 × 10^−1^	1.380 × 10^0^
	5	Mean	**3.052 × 10^1^**	2.772 × 10^1^	2.814 × 10^1^	2.743 × 10^1^	2.846 × 10^1^	2.755 × 10^1^	2.743 × 10^1^	2.821 × 10^1^
		Std	1.276 × 10^−1^	6.629 × 10^−1^	7.081 × 10^−1^	2.033 × 10^−1^	4.726 × 10^−1^	4.307 × 10^−1^	4.056 × 10^−1^	6.071 × 10^−1^
Baseball Diamond	2	Mean	**2.433 × 10^1^**	2.362 × 10^1^	2.394 × 10^1^	2.348 × 10^1^	2.431 × 10^1^	2.404 × 10^1^	2.371 × 10^1^	2.426 × 10^1^
		Std	2.630 × 10^−6^	4.554 × 10^−1^	5.120 × 10^−1^	3.796 × 10^−1^	4.986 × 10^−2^	3.648 × 10^−1^	3.914 × 10^−1^	2.467 × 10^−1^
	3	Mean	**2.667 × 10^1^**	2.534 × 10^1^	2.599 × 10^1^	2.460 × 10^1^	2.658 × 10^1^	2.576 × 10^1^	2.521 × 10^1^	2.656 × 10^1^
		Std	1.937 × 10^−3^	1.081 × 10^0^	1.163 × 10^0^	5.175 × 10^−1^	2.012 × 10^−1^	7.865 × 10^−1^	5.662 × 10^−1^	4.273 × 10^−1^
	4	Mean	**2.869 × 10^1^**	2.629 × 10^1^	2.646 × 10^1^	2.639 × 10^1^	2.827 × 10^1^	2.645 × 10^1^	2.656 × 10^1^	2.712 × 10^1^
		Std	2.231 × 10^−2^	5.187 × 10^−2^	4.646 × 10^−1^	1.404 × 10^−1^	5.352 × 10^−1^	4.283 × 10^−1^	4.504 × 10^−1^	1.065 × 10^0^
	5	Mean	**3.030 × 10^1^**	2.780 × 10^1^	2.780 × 10^1^	2.789 × 10^1^	2.929 × 10^1^	2.780 × 10^1^	2.794 × 10^1^	2.793 × 10^1^
		Std	7.704 × 10^−2^	3.650 × 10^−15^	3.650 × 10^−15^	1.903 × 10^−1^	6.946 × 10^−1^	3.650 × 10^−15^	3.592 × 10^−1^	4.029 × 10^−1^
AvgRank			**1**	6.125	4.5	7.375	2	5.625	6.125	3.25

**Table A7:** Mean, Std, and AvgRank of SSIM for remote sensing images under multi-threshold segmentation.

Image	TH	Type	MALA	ALA	EALA	SCA	GWO	WOA	HHO	MRFO
Beach	2	Mean	**6.944 × 10^−1^**	6.806 × 10^−1^	6.958 × 10^−1^	6.636 × 10^−1^	6.948 × 10^−1^	6.964 × 10^−1^	6.616 × 10^−1^	6.889 × 10^−1^
		Std	2.280 × 10^−16^	4.354 × 10^−2^	5.719 × 10^−3^	4.613 × 10^−2^	2.407 × 10^−3^	3.496 × 10^−2^	5.791 × 10^−2^	2.498 × 10^−2^
	3	Mean	**7.909 × 10^−1^**	7.494 × 10^−1^	7.787 × 10^−1^	6.817 × 10^−1^	7.896 × 10^−1^	7.484 × 10^−1^	6.846 × 10^−1^	7.849 × 10^−1^
		Std	1.139 × 10^−2^	4.720 × 10^−2^	2.434 × 10^−2^	3.605 × 10^−2^	1.785 × 10^−2^	3.425 × 10^−2^	4.398 × 10^−2^	3.226 × 10^−2^
	4	Mean	**8.442 × 10^−1^**	7.764 × 10^−1^	7.904 × 10^−1^	7.301 × 10^−1^	8.001 × 10^−1^	7.773 × 10^−1^	7.493 × 10^−1^	7.958 × 10^−1^
		Std	2.447 × 10^−3^	3.949 × 10^−2^	3.845 × 10^−2^	2.013 × 10^−2^	4.039 × 10^−2^	3.662 × 10^−2^	2.604 × 10^−2^	4.954 × 10^−2^
	5	Mean	**8.787 × 10^−1^**	7.944 × 10^−1^	8.074 × 10^−1^	7.898 × 10^−1^	8.119 × 10^−1^	7.910 × 10^−1^	7.872 × 10^−1^	8.081 × 10^−1^
		Std	7.800 × 10^−3^	2.331 × 10^−2^	2.267 × 10^−2^	1.029 × 10^−2^	1.420 × 10^−2^	1.410 × 10^−2^	1.343 × 10^−2^	2.195 × 10^−2^
Baseball Diamond	2	Mean	**7.440 × 10^−1^**	7.129 × 10^−1^	7.284 × 10^−1^	7.217 × 10^−1^	7.421 × 10^−1^	7.256 × 10^−1^	7.145 × 10^−1^	7.419 × 10^−1^
		Std	1.161 × 10^−3^	2.130 × 10^−2^	1.932 × 10^−2^	2.559 × 10^−2^	3.713 × 10^−3^	2.144 × 10^−2^	3.523 × 10^−2^	9.439 × 10^−3^
	3	Mean	**8.156 × 10^−1^**	7.556 × 10^−1^	7.817 × 10^−1^	7.308 × 10^−1^	8.138 × 10^−1^	7.748 × 10^−1^	7.507 × 10^−1^	8.103 × 10^−1^
		Std	8.856 × 10^−4^	5.661 × 10^−2^	5.614 × 10^−2^	2.798 × 10^−2^	5.121 × 10^−3^	3.885 × 10^−2^	3.128 × 10^−2^	2.093 × 10^−2^
	4	Mean	**8.656 × 10^−1^**	7.819 × 10^−1^	7.878 × 10^−1^	7.869 × 10^−1^	8.510 × 10^−1^	7.871 × 10^−1^	7.914 × 10^−1^	8.115 × 10^−1^
		Std	2.523 × 10^−3^	5.143 × 10^−3^	1.802 × 10^−2^	7.933 × 10^−3^	1.721 × 10^−2^	1.608 × 10^−2^	2.039 × 10^−2^	3.792 × 10^−2^
	5	Mean	**8.956 × 10^−1^**	8.188 × 10^−1^	8.188 × 10^−1^	8.268 × 10^−1^	8.708 × 10^−1^	8.188 × 10^−1^	8.269 × 10^−1^	8.242 × 10^−1^
		Std	2.345 × 10^−3^	1.140 × 10^−16^	1.140 × 10^−16^	1.239 × 10^−2^	2.011 × 10^−2^	1.140 × 10^−16^	1.722 × 10^−2^	1.579 × 10^−2^
AvgRank			**1**	7	4.5	7.25	2	5.75	6.5	3

**Table A8:** Mean, Std, and AvgRank of FSIM for remote sensing images under multi-threshold segmentation.

Image	TH	Type	MALA	ALA	EALA	SCA	GWO	WOA	HHO	MRFO
Beach	2	Mean	8.658 × 10^−1^	8.638 × 10^−1^	**8.664 × 10^−1^**	8.493 × 10^−1^	8.647 × 10^−1^	8.643 × 10^−1^	8.554 × 10^−1^	8.658 × 10^−1^
		Std	2.280 × 10^−16^	6.035 × 10^−3^	2.963 × 10^−3^	1.689 × 10^−2^	1.551 × 10^−3^	2.090 × 10^−2^	3.020 × 10^−2^	3.410 × 10^−4^
	3	Mean	**9.074 × 10^−1^**	8.960 × 10^−1^	8.998 × 10^−1^	8.630 × 10^−1^	9.033 × 10^−1^	8.998 × 10^−1^	8.729 × 10^−1^	9.027 × 10^−1^
		Std	5.228 × 10^−3^	2.944 × 10^−2^	1.914 × 10^−2^	2.793 × 10^−2^	7.722 × 10^−3^	2.380 × 10^−2^	3.068 × 10^−2^	1.229 × 10^−2^
	4	Mean	**9.222 × 10^−1^**	8.840 × 10^−1^	8.927 × 10^−1^	8.875 × 10^−1^	9.133 × 10^−1^	8.941 × 10^−1^	8.802 × 10^−1^	8.992 × 10^−1^
		Std	1.893 × 10^−3^	1.119 × 10^−2^	2.208 × 10^−2^	1.712 × 10^−2^	1.106 × 10^−2^	2.429 × 10^−2^	2.036 × 10^−2^	2.216 × 10^−2^
	5	Mean	**9.404 × 10^−1^**	9.200 × 10^−1^	9.240 × 10^−1^	9.153 × 10^−1^	9.277 × 10^−1^	9.147 × 10^−1^	9.139 × 10^−1^	9.276 × 10^−1^
		Std	4.860 × 10^−3^	1.259 × 10^−2^	1.090 × 10^−2^	6.645 × 10^−3^	7.798 × 10^−3^	5.817 × 10^−3^	6.211 × 10^−3^	8.312 × 10^−3^
Baseball Diamond	2	Mean	7.751 × 10^−1^	7.492 × 10^−1^	7.578 × 10^−1^	7.640 × 10^−1^	**7.756 × 10^−1^**	7.652 × 10^−1^	7.665 × 10^−1^	7.723 × 10^−1^
		Std	1.096 × 10^−3^	2.814 × 10^−2^	2.550 × 10^−2^	4.062 × 10^−2^	1.681 × 10^−3^	2.755 × 10^−2^	1.893 × 10^−2^	1.135 × 10^−2^
	3	Mean	8.283 × 10^−1^	8.257 × 10^−1^	8.284 × 10^−1^	8.170 × 10^−1^	8.243 × 10^−1^	**8.318 × 10^−1^**	8.251 × 10^−1^	8.288 × 10^−1^
		Std	1.282 × 10^−3^	1.502 × 10^−2^	7.722 × 10^−3^	1.969 × 10^−2^	1.109 × 10^−2^	1.521 × 10^−2^	2.551 × 10^−2^	4.515 × 10^−3^
	4	Mean	**8.661 × 10^−1^**	8.246 × 10^−1^	8.319 × 10^−1^	8.285 × 10^−1^	8.624 × 10^−1^	8.305 × 10^−1^	8.382 × 10^−1^	8.449 × 10^−1^
		Std	6.900 × 10^−3^	3.174 × 10^−3^	1.982 × 10^−2^	1.438 × 10^−2^	1.105 × 10^−2^	1.669 × 10^−2^	1.768 × 10^−2^	2.301 × 10^−2^
	5	Mean	**8.931 × 10^−1^**	8.602 × 10^−1^	8.602 × 10^−1^	8.596 × 10^−1^	8.809 × 10^−1^	8.602 × 10^−1^	8.649 × 10^−1^	8.621 × 10^−1^
		Std	4.611 × 10^−3^	1.140 × 10^−16^	1.140 × 10^−16^	1.251 × 10^−2^	2.079 × 10^−2^	1.140 × 10^−16^	1.280 × 10^−2^	8.779 × 10^−3^
AvgRank			**1.625**	6.375	4.5	7.125	2.75	4.875	5.875	3.0
